# The mitochondrial proteome of diplonemids: from conventional pathways to eccentric RNA editing and transcript processing

**DOI:** 10.1186/s12864-025-12233-1

**Published:** 2025-12-11

**Authors:** Michael W. Gray, Matus Valach, Matt Sarrasin, Felix-Antoine Le Sieur, Julius Lukeš, Gertraud Burger

**Affiliations:** 1 Department of Biochemistry and Molecular Biology and Institute for Comparative Genomics, Dalhousie University, Halifax, NS Canada; 2 Département de Biochimie and Robert-Cedergren Center for Bioinformatics and Genomics, Université de Montréal, Montréal, QC Canada; 3 Institute of Parasitology, Biology Centre, Czech Academy of Sciences, České Budějovice (Budweis), Czech Republic; 4 Faculty of Sciences, University of South Bohemia, České Budějovice (Budweis), Czech Republic

**Keywords:** Mitochondria, Proteome, Diplonemids, RNA processing, Paradiplonema

## Abstract

**Background:**

Diplonemids constitute an abundant and geographically widespread but little-studied group of marine protists. A hallmark of this lineage, the kinetoplastid sister group within Euglenozoa, is a mitochondrial genome comprising numerous small circular DNA molecules that carry fragments of mitochondrial genes. Complex RNA processing of the corresponding transcripts involves numerous ligation and RNA editing steps in the production of mature RNA species. To assess the diplonemid mitochondrial proteome and, in particular, to search for proteins that might mediate RNA processing, we undertook a comprehensive in silico analysis to predict candidate mitochondrial proteins in the type species *Diplonema papillatum*.

**Results:**

Using sequence similarity searches in conjunction with a mitochondrial targeting pipeline, we identified at least 1878 candidate nucleus-encoded mitochondrial proteins in addition to 16 mitochondrion-encoded proteins described previously. Despite the highly unconventional nature of the mitochondrial genome in *D. papillatum*, its mitochondrial proteome (mitoproteome) contains virtually all the functionally most important proteins that are ubiquitous among aerobic mitochondria, and several novel proteins that have been recruited in the euglenozoan last common ancestor to augment complexes involved in coupled electron transport oxidative phosphorylation and mitochondrial ribosome formation. Notably, we identified several individual proteins and multi-protein families that are candidates for RNA ligation and editing enzymes.

**Conclusions:**

This first comprehensive mitoproteome data for a diplonemid, together with published mitoproteome data for other members of Discoba, allows us to make inferences about marked changes in mitochondrial structure and function that have occurred since the divergence of diplonemids and other euglenozoans from the last common discobid ancestor.

**Supplementary Information:**

The online version contains supplementary material available at 10.1186/s12864-025-12233-1.

## Introduction

Diplonemid flagellates are among the most diverse and abundant of known marine microbial eukaryotes, exhibiting a wide range of habitats [1, 2]. Together with euglenids and kinetoplastids, diplonemids comprise Euglenozoa, a phylum within the eukaryotic supergroup Discoba [3]. A notable feature of Discoba is the exceptional diversity in the organization, gene content and mode of expression of the mitochondrial genomes of its constituent clades. At one extreme, the discobid group of jakobid flagellates contains the least derived, most bacteria-like and most gene-rich mitochondrial DNAs (mtDNAs) so far found anywhere among eukaryotes [4, 5]. Conversely, Discoba also includes clades that lack conventional mitochondria and mtDNAs, containing instead mitochondrion-related organelles (MROs), structurally and functionally reduced mitochondria harboring highly reduced mtDNA, or no mitochondrial genome at all [6].

Between these two extremes are discobid protists whose mtDNAs display a bewildering divergence in physical organization and functional expression [7]. Diplonemids are particularly notable in this regard, with the single mitochondrion of the model species *Diplonema papillatum* containing ~ 260 Mbp, the highest amount of DNA documented so far in any organelle [8]. Studies in *D. papillatum* have revealed a mitochondrial genome comprising a large array of small circular DNA molecules, with fragmented protein-coding and rRNA genes whose coding sequences are distributed across multiple mtDNA circles [9]. Transcription yields primary products that undergo extensive RNA processing to generate mature mRNAs and rRNAs. This processing includes insertion (U-addition) as well as substitution (A-to-I and C-to-U) editing followed by correctly ordered ligation of multiple subgenic primary transcripts [7, 10–12]. Extremely truncated small subunit (SSU) and large subunit (LSU) rRNAs are also encoded by and transcribed from mitochondrion-specific small DNA circles, and are assembled with a large cohort of nucleus-encoded mitoproteins to form the most protein-rich mitoribosome yet described [13, 14]. Very different but equally unconventional mitochondrial genomes are found in kinetoplastids [15], the sister group of diplonemids, as well as in the earlier-branching euglenids [16].

The exceptional mitochondrial genome diversity within Discoba raises the issue of the nature of their mitochondrial proteins (mitoproteome), and the extent to which mitoprotein composition, both nuclear DNA- and mtDNA-encoded, mirrors this diversity. In the case of the jakobid flagellate *Andalucia godoyi*, whose mtDNA is strikingly bacteria-like, a study of its predicted mitoproteome revealed an array of retained bacteria-like traits that have evidently been lost from, or individually show a punctuate distribution in, other eukaryotes [17]. Broader comparative mitoproteome studies throughout eukaryotes, especially eukaryotic microbes (protists) [18–23] have revealed a consistent pattern of a limited number of well conserved functions underpinned by mitoproteins that trace their ancestry to the last eukaryotic common ancestor (LECA), with clade-specific and species-specific proteins/functions superimposed on the foregoing conserved class. Further extending these comparative mitoproteome studies provides a valuable complement to our extensive knowledge on mitochondrial genome diversity and promises to further enhance our overall understanding of mitochondrial evolution and function.

Our recent publication of the nuclear genome sequence of *D. papillatum* [24] allows us to assess the mitoproteome in this ecologically important protist. In particular, having an inventory of robustly inferred mitoproteins provides us an opportunity to identify candidates involved in the extensive RNA processing that mitochondrial transcripts undergo in this organism. Accordingly, we carried out an in silico analysis of the *D. papillatum* proteome to catalog proteins most likely targeted to mitochondria, also using mass spectrometry of isolated mitochondria or mitoprotein complexes to strengthen mitochondrial localization assignments. Of special interest with regard to potential mitochondrial RNA processing candidates were a number of mitoprotein classes, including pentatricopeptide repeat (PPR) proteins, DEAD/DExH box helicases and DnaJ domain-containing proteins. The results summarized here provide the basis for further investigation of the biochemistry of mitochondria and especially mitochondrial RNA processing not only in diplonemids, but in eukaryotes in general.

## Results

### Identification of candidate mitoproteins

The *D. papillatum* proteome was inferred from the nuclear genome sequence [24] and deposited in NCBI’s Bioproject 883,718. For the present study, the data were further enhanced by expert curation, mainly removing gene models that included repetitive elements and lacked evidence for transcription (see Materials and Methods). We used a combination of BLAST and domain- or protein-specific profile HMM searches and mitochondrial targeting algorithms to select candidate mitoproteins from the complete *D. papillatum* proteome. As a source of search queries, we focused particularly on other members of Discoba, including *A. godoyi* as a representative of the likely ancestral state of this lineage, and kinetoplastids, especially *Trypanosoma brucei*, as the closest evolutionary relatives of diplonemids.

As detailed in Materials and Methods, we implemented an objective screening procedure to assess the likelihood of mitochondrial localization predicted by various targeting methodologies. This screen resulted in almost 6000 entries with some degree of predicted mitochondrial localization (Supplementary Table S1) out of a total of over 37,000 inferred protein-coding genes in the *D. papillatum* genome [24]. We divided candidates into four classes, based on likelihood of mitochondrial localization: (1) almost certainly mitochondrial, weighted average (WA) score > 0.75; (2) very likely mitochondrial, WA = 0.5–0.75; (3) likely mitochondrial, WA = 0.2–0.5; and (4) low probability of mitochondrial localization, WA = < 0.2. Proteins having a WA score of 0 were classified as non-mitochondrial.

For the purposes of this study, with few exceptions we selected only entries in categories 1 and 2 as candidate mitoproteins (Supplementary Table S2). Significantly, using as queries known mitoproteins from other organisms, the vast majority of predicted *Diplonema* proteins retrieved by BLAST searches fall into categories 1 or 2. However, we have included in our list a small number of proteins having scores < 0.5, as well as a few entries with a score of 0; in these cases, we considered other evidence, including direct isolation from purified *Diplonema* mitochondria or sub-mitochondrial fractions and/or exclusive mitochondrial localization and function in other organisms.

**Fig. 1:**
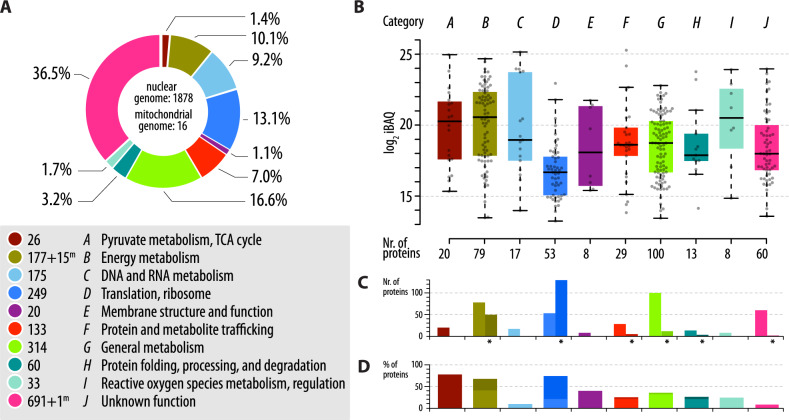
Functional categories comprising the *D. papillatum* mitoproteome.** A** Numbers in each of ten functional categories (A–J) are for nucleus-encoded (1878 total) and for mitochondrion-encoded (16 total, ‘m’ superscript) mitoproteins. Percentages are based on all mitoproteins (1894 total). **B** Abundance of proteins in a given category that were reliably detected by mass spectrometry in *Diplonema*’s mitochondrial fractions [26]. Box limits indicate the 25th and 75th percentiles and their centre lines show the medians. Each dot represents a protein’s log2 iBAQ value (average from four replicates). **C** Number of proteins in each category detected by mass spectrometry in the mitochondrial fraction (same as in B, bars in lighter shades) and in additional *Diplonema* proteomics datasets, e.g., mitoribosome or respiratory chain complexes (bars in darker shades and indicated by asterisks; see Methods for details). **D** Percentage of proteins in each category detected in experiments shown in **C** (cumulative bars)

In this way, we predict a *Diplonema* mitoproteome of at least 1878 unique-sequence, nucleus-encoded proteins, 97% of which have a WA score > 0.5, in addition to 16 mtDNA-encoded proteins identified previously [11, 25] (Supplementary Table S2, tab K, Statistics). Allied transcriptomic data verify that these predicted proteins are indeed expressed and have been manually curated. For purposes of comparison, these candidates have been sorted into the functional categories used in other recent mitoproteome studies [17, 21], with the distribution of proteins in these categories listed in Fig. 1 and Supplementary Table S2 (tab K). Compared with the predicted *Andalucia* mitoproteome, several categories are over-represented in *Diplonema*. In particular, expansion of DNA and RNA Metabolism (category C) and Protein Folding (category H) reflects the presence of multiple members of specific functional families that may participate in the unusual RNA metabolism that occurs in *Diplonema* mitochondria, as discussed below. Over one-third of the candidate *Diplonema* mitoproteins are functionally uncharacterized. Proteomics experiments confirmed the enrichment of 20.6% of the predicted proteins in the sub-cellular fraction containing mitochondria [26] and more targeted approaches revealed an additional 10.6% in mitochondrial protein and ribonucleoprotein complexes [14, 27] (Fig. 1B–D).

### Overview of the predicted *Diplonema* mitoproteome

Here we present a succinct summary of notable aspects of the predicted *Diplonema* mitoproteome (Supplementary Table S2), before turning to a detailed analysis of protein sets of particular interest in the context of mitochondrial RNA metabolism.

#### Pyruvate metabolism, TCA cycle

*Diplonema* lacks a conventional E1-E2-E3 mitochondrial pyruvate dehydrogenase (PDH), employing instead an archaeal type E1 subunit (AceE), likely in conjunction with an E2 subunit from either the branched-chain ketoacid dehydrogenase (BCKDH) complex or the 2-oxoglutarate dehydrogenase (OGDH) complex, as well as a single E3 subunit presumed to function in the latter two complexes [26]. *Diplonema* has in addition a mitochondrial pyruvate: ferredoxin oxidoreductase (PFO), which presumably could be employed under anaerobic conditions to generate acetyl-CoA and reduced ferredoxin. Curiously, *Diplonema* encodes at least six mitochondrial PDH kinases, compared with a single one in *Andalucia* and only two homologous sequences in *T. brucei.*

A full set of TCA cycle enzymes is present [28]. Two aconitase entries provide a good example of gene duplication followed by differential subcellular localization of the protein products, with mitochondrion-targeted DIPPA_23444 (WA score = 0.871) having a 45 N-terminal extension relative to non-mitochondrial DIPPA_07820 (WA score = 0). The two proteins are clearly paralogous (85.5% sequence identity over the shared region), with their intron-less genes located on different genomic scaffolds.

#### Energy metabolism

The *Diplonema* electron transport chain (ETC) complexes CI – CIV and ATP synthase CV constitute a mix of conventional subunits (ones widely conserved and distributed among eukaryotes) as well as novel, lineage-specific subunits, some of which have homologs in other euglenozoans, others apparently limited to diplonemids. As indicated in Supplementary Table S2, several of the components in this category are multi-functional, involved in other mitochondrial pathways as well as energy metabolism.

### Conventional subunits

Most of the expected conventional components of complexes CI – CV are present in the predicted *Diplonema* mitoproteome, although as indicated in Supplementary Table S2, some components were not retrieved in our search and so are considered to be absent or so diverged in sequence that our procedure failed to recognize them. Selected aspects of each *Diplonema* complex are summarized below.

#### Complex I (CI)

Except for NDUFS4 (also known is AQDQ), all expected components (17) of the α-proteobacterial CI core are present. Nad1 to Nad9 and Nad4L are encoded in *Diplonema* mtDNA and were previously identified [27]. *Diplonema* NDUFS1, also known as Nad11, corresponds to the N-terminal one-third of a conventional NDUFS1 (as encoded in *Andalucia* mtDNA), consisting of the ferredoxin domain that is deeply embedded in the matrix portion of the CI 3D structure. The ‘missing’ C-terminal portion corresponds to the molybdopterin-binding domain, which normally protrudes from CI. A C-terminal truncated protein is a feature of Euglenozoa, as trypanosomatid NDUFS1 sequences (at 250–300 aa) and *Euglena gracilis* NDUFS1 (385 aa) are similarly short. An analogous situation has been described in certain brown algae [29], except in that case the truncated NDUFS1 is encoded in the mitochondrial rather than the nuclear genome. As we did not retrieve a candidate corresponding to the NDUFS1 C-terminal region, it appears that this portion of the protein is missing or substituted by a different protein. The latter possibility is the case in *Euglena*, as the recently determined structure of its CI shows that this role is fulfilled by a newly recruited protein with little sequence but some structural similarity to the NDUFS1 C-terminal portion [30]. As we could not identify any sequence or structural diplonemid counterpart of this protein, it seems likely that *Diplonema* has settled on a different solution.

Of 15 CI proteins designated as ‘eukaryote-specific’ [31], all of which are present in *Andalucia*, three appear to be missing in *Diplonema*: NDUFA1, NDUFA11 and NDUFB3 (also known as MWFE, B14.7 and B12, respectively). As in *Andalucia*, two sequence-divergent versions of NDUFA9 (39-kDa subunit) are present in *Diplonema*. In BLASTp searches, each *Andalucia* protein specifically retrieves one of the two *Diplonema* homologs, DIPPA_20257 and DIPPA_34793. The latter appears to be the homolog of a ‘unique’ trypanosomatid CI accessory subunit, NDUTB5 (see below). Notably, *Diplonema* contains no obvious counterparts of the 15 subunits designated ‘metazoan-specific’ by [31], whereas *Andalucia* has seven.

As in other non-opisthokont eukaryotes (all except animals and fungi), *Diplonema* CI contains two γ carbonic anhydrase (γCA) homologs, designated CA9 (CA) and CA9-like (CAL) [27]. In addition, four other mitochondrion-targeted carbonic anhydrase family proteins are present. Neither NUUM nor NUXM, two ‘fungal-specific’ proteins, is present in *Diplonema* (*Andalucia* has homologs of both).

#### Complexes II - IV

We identified candidates corresponding to conventional CII subunits Sdh1 to Sdh4. As in *Euglena* [32] and trypanosomatids [33], the nuclear Sdh2 gene is split, giving rise to separate proteins, Sdh2_N_ and Sdh2_C_, which correspond to the two halves of a conventional Sdh2 subunit.

CIII subunits α and β of the matrix processing peptidase, apocytochrome *b* (Cob; mtDNA-encoded), Rieske iron-sulfur protein, cytochrome *c*_1_ (Cyc1) and subunits Qcr6, Qcr7, Qcr8 and Qcr9 were all identified. Qcr10 [34] was not retrieved.

Cox1, Cox2 and Cox3 subunits of CIV are mtDNA-encoded in *Diplonema*. Cox1 in *Diplonema*, *Euglena* and *Trypanosoma* appear to lack a conserved, functionally important C-terminal motif. In other organisms whose mtDNA-encoded Cox1 also lacks this motif, it is present instead in a nucleus-encoded, imported protein, Cox1_C_ [35]; however, we did not retrieve a homologous *Diplonema* protein. Of seven additional CIV subunits identified in *Andalucia*, only three were recovered in *Diplonema* (Cox4, Cox5b, Cox6b/Cox12).

#### Complex V (CV)

A recent comprehensive comparative study concluded that a canonical set of 17 core proteins, which make up the mitochondrial F_1_F_o_ ATP synthase in fungi and animals, is in fact ancestral to all eukaryotic CVs, present in the last eukaryotic common ancestor (LECA) [36]. This core set is composed of five soluble F_1_ subunits (α, β, γ, δ, ε) and a membrane-bound F_o_ sector comprising the peripheral stalk (subunits OSCP, 8, b, h, d) and proton half-channels (a, c). The remaining subunits (f, i/j, e, g, k) are involved in dimer formation, which does not occur in bacteria or chloroplasts.

We identified in the *D. papillatum* mitoproteome candidates for almost the entire core set of CV subunits, with subunit a (Atp6) being mtDNA-encoded; the only exception is subunit h, which is apparently absent from all euglenozoans [36, 37]. We also identified two variant homologs of novel *T. brucei* subunit p18 [38], an additional F_1_ subunit that interacts with the α subunit [39].

Multiplicity of genes encoding various subunits is a notable feature of *Diplonema* ATP synthase. For example, triplicate, non-identical Atp1 (α) genes, DIPPA_18956, DIPPA_04057 and DIPPA_03046, are found on separate scaffolds. Each gene has a single, differently-sized intron (880, 5665, 4244 bp, respectively) in the same position, with amino acid substitutions mostly confined to the N-terminal 70 (corresponding to the mitochondrial presquence) and C-terminal 100 residues. Atp2 (β), Atp4 (b), Atp9 (c) and Atp15 (ε) are other ATP synthase subunits encoded by multiple non-identical genes. In proteomics experiments, we observed peptides originating from most of the different paralogs of a given protein, with isoform abundances sometimes varying (e.g., Atp4 variant DIPPA_25906 appeared more abundant than DIPPA_22523).

### Novel euglenozoan- and diplonemid-specific subunits

A characteristic feature of kinetoplastid respiratory complexes is the presence of novel subunits [33, 38, 40–42]. Table 1 lists the *T. brucei* versions of these additional subunits, and the corresponding homologs we retrieved in *D*. *papillatum*. Homologs of some of the *T. brucei* proteins have also been identified in *E. gracilis* [43, 44], indicating that recruitment of additional subunits to the individual respiratory complexes was already underway in the last euglenozoan common ancestor.

Because homologs of these novel *T. brucei* proteins are poorly conserved in sequence across kinetoplastids, it is possible that at least some of those that appear to be missing in *Diplonema* are simply too divergent to be identified. Alternatively, lineage-specific proteins may substitute in some cases. In this regard, proteomics analysis of isolated *Diplonema* CI has identified more than a dozen novel subunits that appear to be diplonemid-specific (i.e., present only in diplonemid species but not euglenids or kinetoplastids) [27], in addition to the euglenozoan-specific subunits listed in Table 1.

**Table 1:** Euglenozoan-Specific proteins in complexes I to V

	Name	Trypanosoma^a^	Diplonema^a^	Euglena
CI	NDUTB1	XP_803505.1	X ^b^	
	NDUTB2	XP_827075.1	DIPPA_10860	NDUEG3
	NDUTB3	XP_846361.1	DIPPA_33120	NDUEG5
	NDUTB4	XP_827174.1	DIPPA_33245	
	NDUTB5 (NDUFA9 homolog)	XP_827678.1	DIPPA_34793	NDUEG4
	NDUTB6	XP_827669.1	DIPPA_32547	
	NDUTB7	XP_822995.1	X	
	NDUTB8	XP_822477.1	DIPPA_15903	
	NDUTB9	XP_829447.1	X	
	NDUTB10^c^	XP_829639.1	DIPPA_00395^c^	
	NDUTB11^c^	XP_845007.1	DIPPA_27907^c^	
	NDUTB12	XP_828770.1	DIPPA_18104	NDUEG2
	NDUTB13	XP_001218776.1	X	
	NDUTB14	XP_951631.1	X	
	NDUTB15	XP_843958.1	X	
	NDUTB16	XP_845263.1	X	
	NDUTB17	XP_845322.1	DIPPA_02911^d^	NDUEG1
	NDUTB18	XP_828525.1	DIPPA_02008	
	NDUTB19	XP_846353.1	DIPPA_01215	
	NDUTB20	XP_847256.1	X	
	NDUTB21	XP_828435.1	X	
	NDUTB22	XP_803433.1	X	
	NDUTB23	XP_827034.1	X	
	NDUTB24	XP_827273.1	X	
	NDUTB25	XP_827651.1	X	
	NDUTB26	XP_822374.1	X	
	NDUTB27	XP_951505.1	?^e^	
	NDUTB28	XP_844580.1	X	
	NDUTB29	XP_846014.1	DIPPA_30123	
	NDUTB30	XP_847086.1	DIPPA_07751	NDUEG11
	NDUTB31	XP_847386.1	DIPPA_70128	
CII	SDH5	XP_843938.1	DIPPA_11439	
	SDH6	XP_847394.1	DIPPA_11233	
	SDH7	XP_845370.1	DIPPA_32725	
	SDH8	XP_951654.1	DIPPA_34914	
	SDH9	XP_822557.1	DIPPA_02316	
	SDH10	XP_844725.1	DIPPA_29202	
	SDH11	XP_847519.1	DIPPA_17125	
CIV	COXIV (Tb927.1.4100)	XP_001219104.1	DIPPA_22841	COXEG6
	COXV (Tb927.9.3170)	XP_803621.1	DIPPA_06444	COXEG7
	COXVI_1 (Tb927.10.280)	XP_822288.1	DIPPA_03634	COX6B
	COXVI_2 (Tb927.10.280)	XP_822288.1	DIPPA_23344	COX6B
	COXVII (Tb927.3.1410)	XP_843735.1	DIPPA_28784	
	COXVIII (Tb927.4.4620)	XP_844611.1	X	
	COXIX (Tb927.10.8320)	XP_823066.1	X	
	COXX (Tb11.01.4702)	XP_829371.1	X	
	P27 (Tb11.0400)	CAJ17015.1	X	
	Tb927.10.4880	XP_822735.1	DIPPA_21643	COX5B-2
	Tb09.211.1900	XP_846319.1	X	
	Tb09.211.4740	XP_827614.1	DIPPA_34859	
	Tb10.70.1890	XP_822767.1	DIPPA_19858	
	Tb927.7.6990	XP_846319.1	DIPPA_15091	
	Tb11.01.1900	XP_011780284.1	DIPPA_23802	
	Tb11.46.0006	XP_828285.1	DIPPA_24645	
	Tb927.11.4870	XP_828562.1	DIPPA_18591	
	Tb09.211.4400	XP_827579.1	DIPPA_34751	
	Tb927.8.6080	XP_847438.1	DIPPA_05568	
CV	ATPTB1	XP_822310.1	DIPPA_30843	6TDV|a
	ATPTB3	XP_828699.1	DIPPA_23529	6TDV|b
	ATPTB4	XP_823205.1	DIPPA_16978	6TDW|C
	ATPTB6	XP_828218.1	DIPPA_02896	6TDV|d
	ATPTB11	XP_843812.1	DIPPA_06559	
	ATPTB12	XP_844981.1	DIPPA_23443	6TDV|e
	ATPTB14	XP_847163.1	X	
	p18	XP_067082445	DIPPA_22523	

^a^ Sequences are from *Trypanosoma brucei brucei* TREU927 and *Diplonema papillatum* (ATCC50162)

^b^ X denotes that a homolog was not retrieved from *D. papillatum* or any of 11 other diplonemids (see Methods for the full list)

^c^ Orthology assignment tentative: several closely related *Diplonema* sequences retrieved. The listed *Diplonema* homolog has the lowest BLASTp E value

^d^ Bifunctional protein in *Diplonema*. Listed as “mitochondrial ribosomal protein L69 (mL69)” in category D

^e^ PF00160.21 domain (Pro_isomerase); large number of homologs retrieved: orthology assignment not possible

### Assembly proteins

We identified homologs of known assembly factors for each of the ETC complexes, although several expected factors were not retrieved (CI DMAC1; CII SdhAF3; CIII UQCC2/Cbp6 and UQCC3/Cbp4; CIV Cmc2, Coa4, Coa6, Cox16, Cox18, Cox20). Numerous factors are present in multiple distinct copies, including three NDUFAF7/MidA (CI), three Bcs1 (CIII), and three Sco1/Sco2 and two Shy1/Surf1 (CIV).

Several CIV assembly proteins (as well as certain ETC subunits) are characterized by a twin Cx_9_C motif [45] and adopt a coiled-coil-helix–coiled-coil-helix (CHCH) domain. Yeast (*Saccharomyces cerevisiae*) contains 17 twin Cx_9_C proteins whereas 29 have been identified in humans (11 ETC subunits and 12 involved in CIV assembly). To date, we have found 22 such proteins in the *Diplonema* mitoproteome, all < 260 amino acids long, including five CIV assembly proteins, six ETC subunits and nine additional proteins that we have so far not been able to assign (Table 2). Some of these nine uncharacterized twin Cx_9_C proteins may represent highly diverged ETC subunits or CIV assembly factors that we failed to detect in our similarity searches, or novel ETC proteins (e.g., CI NDUDP1).

**Table 2:** Cx_9_C motif proteins in *Diplonema papillatum*

Function	Assignment	DIPPA_	AA	Motifs	Sequence^a^
ETC Subunits	NDUFS5 (CI)	21368	165	2	… CHMFKRSFNLC - [13] - CAIEKEDWYNC …
	NDUFA8 (CI)	34842	237	3	… CKVMKQNGNLC - [21] - CPKQTVGWVKC - [10] - CRDLQVEWESC …
	NDUFB7 (CI)	01509	254	2	… CSHKYIPLMLC - [14] - CKNHAHEYELC …
	NDUP1 (CI)	12325	322	2	… CIAGGSGITPC - [84] - CGPDGLMDAVC …
	Qcr6 (CIII)	27291	111	2	… CQQMKAEYESC - [11] - CAYQYQNMWGC …
	Cox6b/Cox12 (CIV)	06841	86	2^b^	… CYQAKDDYYKC - [7] - CSKEIEGYETTC …
ETC Assembly	Coa5/Pet191 (CIV)	17221	157	3	… CYNIRELYIQC -[5] - CIREKKDFEAC - [5] - CMAERRGLSQC …
	Cmc1 (CIV)	11083	168	2	… CRPHHEEVISC - [10] - CKPLLSEYYTC …
	Cox17 (CIV)	22952	95	2	… CPSTRSARDEC - [8] - CKSQIEAHYQC …
	Cox19 (CIV)	09793	123	2	… CRGTIEEYFRC - [10] - CREEARTYLRC …
	Cox23 (CIV)	10484	136	4	… CHPAMTATRQC - [10] - CIRAESEARSC - [14] - CKREYSAWATC - [17] - CFVERSQVDTC …
Ribosomal Proteins	mS37	34165	238	2	… CTGAFQHVVKC - [15] - CAQELSNYFQC …
Cardiolipin Synthesis	Mdm35	35039	77	2	… CQKEAKVYAQC - [14] - CESEFRQYRAC …
Unknown		22896	72	2	… CHPVSEKFFTC - [22] - CRGELEAYKKC …
		01507	126	2	… CNRLYHVAVKC - [11] - CKPEMNEIVAC …
		18076	157	2	… CMEVDKAFHQC - [10] - CQIVFADLTSC …
		30635	160	2	… CQQYDTAFHQC - [10] - CKNAVRGALPC …
		33142	134	2	… CPLEYRALVKC - [7] - CSQEQTGFSRC …
		50397^c^ ^62583c^	105	2	… CSCIRLYPRLC - [38] - CARRDSTELFC …
		19780	109	2	… CPEEKERVVSC - [14] - CSQEVKKFSQC …
		04955	162	2	… CEEKWKDYLRC - [11] - CVAIREVADVC …
		11083	223	2	… CRPHHEEVISC - [10] - CKPLLSEYYTC …

^a^ Cx_9_C motifs are shown, with numbers in square brackets indicating the number of amino acid residues separating them

^b^ Cx_9_C/Cx_10_C, characteristic of Cox12 proteins

^c^ Duplicate identical sequences

### Mitochondrial LYR (leucine/tyrosine/arginine) proteins

Proteins in this category contain a Complex1_LYR (PF05347) motif and are implicated in various mitochondrial functions from ETC complex biogenesis to acetate metabolism [46]. We list six proteins of unknown function in this category. Additional LYRM proteins of known function identified in the present study include CI subunit NDUFB9, CI assembly protein NDUFA6, CIII assembly protein MZM1L/LYRM7, electron transfer flavoprotein regulatory protein ETFRF1/LYR5, mitochondrial LSU assembly protein mt-LAF10, and iron-sulfur cluster biosynthesis protein Isd11 (see Supplementary Table S2, categories B, D and G).

### Alternative respiratory pathway

An alternative respiratory pathway comprising alternative oxidase (AOX), a rotenone-insensitive (type II) NADH dehydrogenase and glycerol 3-phosphate dehydrogenase is present in *T. brucei* mitochondria, is essential in the bloodstream stage of the organism, and functions in other phases of its life cycle [47–49]. All three components of this pathway are predicted to be present in the *Diplonema* mitoproteome. As in *Andalucia*, we identified two distinct alternative oxidases, AOX_1 (DIPPA_09286) and AOX_2 (DIPPA_09698), the latter represented by three isoforms differing slightly in sequence and encoded on a different genomic scaffold from that encoding AOX_1.

Downstream of the AOX_1 gene, we identified fused ORFs that appear to specify a split AOX, with N-terminal and C-terminal halves represented by separate entries: DIPPA_09289, AOX_N_ and DIPPA_09287, AOX_C_, respectively, both mitochondrion-targeted. Curiously, the predicted AUG initiation codon for the AOX_C_ gene directly abuts the UAG termination codon for the AOX_N_ gene. Based on sequence similarity and alignment considerations, we surmise that this peculiar arrangement likely arose by AOX gene duplication followed by an AAG (K) to UAG (termination) mutation. Given that expression of the AOX_N_/AOX_C_ ‘half genes’ is < 1% that of the AOX_1 gene, it may be that the two half-AOX genes are not really functionally important, but simply remnants of a duplicated region on a deteriorating evolutionary path. We note that there is no evidence for analogous half-AOX genes in other diplonemids. On the other hand, the fact that these half-genes are transcribed at all does raise the possibility of their translation.

### Electron transfer flavoprotein complex (ETFC)

This complex is located on the matrix side of the inner mitochondrial membrane and transfers electrons from primary dehydrogenases to terminal respiratory acceptors such as electron-transferring flavoprotein dehydrogenase, primarily in the fatty acid-oxidation pathway. We identified ETF α and β subunits as well as an ETF-ubiquinone oxidoreductase and ETF flavoprotein regulatory factor 1 (ETFRF1/LYR5), confirming that this broadly distributed system operates in *Diplonema* mitochondria.

### DNA & RNA metabolism

#### DNA metabolism

Recent studies have considerably expanded the inventory of mitochondrial DNA polymerases (mt-DNAPs) in Discoba and clarified their evolution [50–52]. Two types of family A mt-DNAPs, having different evolutionary origins, occur specifically in Euglenozoa. The ancestral mitochondrial PolIA appears to have been derived from a non-mitochondrial Pol*θ* homolog re-targeted to mitochondria, whereas PolIBCD+ (restricted to diplonemids and kinetoplastids) has been postulated to have arisen *via* lateral gene transfer from a single autographivirus family A DNAP [50]. These two mt-DNAP types differ from a novel discobid family A DNAP, termed rdxPolIA, initially reported in *A. godoyi* [17] and subsequently identified throughout the lineages Discoba, Ancyromonadida and Malawimonadida [52]. rdxPolA has been proposed as the direct descendant of the PolI in the α-proteobacterial endosymbiont that gave rise to the mitochondrion [52].

A single PolIA and three distinct PolIBCD+ DNAPs have been characterized in *T. brucei* mitochondria [53]. The PolIA homolog in *D. papillatum* (DIPPA_23022) appears to be targeted to the nucleus as it has a WA score = 0 (non-mitochondrial). In contrast to the multiple PolIBCD+ DNAPs in *T. brucei* mitochondria, *D. papillatum* has just one (DIPPA_22214). *T. brucei* also possesses a mt-DNAP beta [53] but again the *Diplonema* homolog (DIPPA_01172) scores as non-mitochondrial; *Diplonema* has instead a different mt-DNAP X/beta (DIPPA_70088), but lack of conserved catalytic residues indicates that the protein has a role other than replicating or repairing DNA. Finally, *Diplonema* has a mt-DNAP IV/kappa, a homolog of which has previously been characterized in *Trypanosoma cruzi* mitochondria [54]. Other proteins expected to be involved in DNA replication, recombination and repair in *Diplonema* mitochondria include a free-standing 5′-to-3′-exonuclease and several 3′-to-5′-exonucleases, helicases, topoisomerases I and II and endonucleases, including two TatD-related DNases.

Notably, *Diplonema* encodes an unusual 21-member family of related mitochondrion-targeted proteins in which two amino acids, alanine and lysine, comprise > 60% of these proteins, with three-quarters of them having a C-terminal high mobility group (HMG) motif similar to that in HMG-box protein DIPPA_32963. Given that HMG-boxes are DNA binding domains, and considering the multi-partite physical form of the diplonemid mitochondrial genome, which is made up of numerous small circular molecules [55, 56], it is tempting to suggest that this set of proteins is involved in some aspect(s) of replication and/or maintenance of this unconventional mtDNA. We found a comparable number of proteins with the same C-terminal ~ 80 amino acid-long motif across various diplonemids, further highlighting the potential significance of this protein family. Four additional alanine + lysine-rich proteins are members of the Linker histone H1/H5 (IPR005819) family, which also might suggest a role in *Diplonema* mtDNA metabolism.

#### RNA metabolism

As in all other eukaryotes characterized to date except for jakobids, *Diplonema* uses for mitochondrial gene expression a nucleus-encoded, single-subunit phage T3/T7-like RNA polymerase. We found no evidence in genomic or transcriptomic data of sequences homologous to *Andalucia* mtDNA-encoded bacteria-like RNA polymerase subunits (RpoA to D).

The *Diplonema* mitoproteome contains at least 13 RNA helicases, three of which are implicated in LSU assembly (and listed in category D). Furthermore, at least eight RNases of various types were identified, but none with activities involved in tRNA biosynthesis, such as RNase Z and CCA tRNA nucleotidyltransferase (3′-end maturation). This absence is not surprising in that no mtDNA-encoded tRNA genes have been identified in *Diplonema*, so that all tRNAs involved in mitochondrial translation must be imported from the cytosol into mitochondria, as in kinetoplastids [57–59]. In fact, *T. brucei* tRNAs imported into mitochondria in vivo are all aminoacylated [60], and we expect that this is also the case in *Diplonema*. 

##### RNA maturation of mitochondrial transcripts

As discussed in detail below, we have identified various RNases, as well as a number of proteins of particular interest in the context of the extensive RNA processing that occurs during the maturation of mitochondrial transcripts. A set of seven small RNA/DNA binding proteins containing a cold shock domain (CSD) are among these potential RNA processing activities.

PPR proteins bind RNA and function in various aspects of RNA metabolism in mitochondria, particularly RNA editing [61]. In the *Diplonema* proteome, we identified 121 unique protein sequences having one or more PPR [62] domains. Most of these PPR proteins (108; 89%) are predicted to be targeted to mitochondria (categories C and D). This very large repertoire is unprecedented for a protist and more in line with the numbers seen in land plant mitochondria, where they were first discovered [63]. Most of the PPR proteins identified here are functionally uncharacterized, although eight have been found to be involved in mitoribosome assembly and structure (category D) [14]. As discussed in detail below, we consider the numerous PPR proteins in the *Diplonema* mitoproteome to be prime candidates for factors involved in RNA ligation and editing during maturation of mitochondrial transcripts.

### Translation, ribosome

*Diplonema* encodes an expected array of mitochondrial translation initiation factors (IF2, IF3), elongation factors (EF-G, EF-Tu, EF-Ts), ribosome release/recycling factors and peptidyl-tRNA hydrolase. Several of these factors (IF2, IF3) play additional roles in ribosome biogenesis. Only a single mitochondrial peptide chain release factor (mt-RF1) was identified, homologous to bacterial RF1, which recognizes UAA and UAG termination codons. Because the third standard termination codon, UGA, has been reassigned to tryptophan in *Diplonema* mitochondria [25], there is no need for a mitochondrial UAA/UGA-decoding RF2 homolog.

Surprisingly, we identified only three mitochondrion-targeted aminoacyl-tRNA synthases (AARSs), distinct from their cytosolic counterparts and specific for aspartate (AspRS), methionine (MetRS) and tryptophan (TrpRS). Table 3 lists all of the AARS sequences found in the *Diplonema* proteome, with a single (cytosolic) synthase detected for most amino acids. The *Diplonema* situation mirrors that in kinetoplastids: in *T. brucei*, all AARSs are single-copy except for AspRS, LysRS and TrpRS [64–66]. One copy of each of these three AARSs is imported into and functions in the *T. brucei* mitochondrion. Thus, mitochondrial translation must use imported cytosolic-type AARSs for the majority of amino acids, which is consistent with the fact that all tRNAs required for mitochondrial translation in kinetoplastids and diplonemids are cytosolic-type and imported from the cytosol. Because these imported cytosolic AARSs lack a classical mitochondrial targeting sequence, how they get into mitochondria remains a mystery. Notably, the diplonemid-kinetoplastid situation contrasts markedly with that in *E. gracilis*, where mitochondrion-targeted AARSs have been identified for all amino acids except arginine and tryptophan [22]. AARS-associated proteins identified in *Diplonema* include Met-tRNA formyltransferase, peptide deformylase and subunit A (GatA) of Glu-tRNA(Gln) amidotransferase; GatB and GatC subunits were not retrieved.

**Table 3:** *D. papillatum* Aminoacyl-tRNA synthases (AARS)^a^

AARS	Mitochondrial	WA Score	Cytoplasmic	WA Score
Ala	—	—	DIPPA_17145.mRNA.1	0
Arg	—	—	DIPPA_12590.mRNA.1, (DIPPA_12590.mRNA.2)	0
Asn	—	—	DIPPA_00466.mRNA.1	0
Asp	DIPPA_05087.mRNA.1	0.882	DIPPA_04483.mRNA.1, DIPPA_02560.mRNA.1	0
Cys	—	—	DIPPA_08482.mRNA.1, DIPPA_18035.mRNA.1	0
Glu	—	—	DIPPA_24500.mRNA.1	0
Gln	—	—	DIPPA_21828.mRNA.1	0
Gly	—	—	DIPPA_11895.mRNA.1	0
His	—	—	DIPPA_22021.mRNA.1, DIPPA_19512.mRNA.1,2	0
Ile	—	—	DIPPA_11435.mRNA.1	0
Leu	—	—	DIPPA_34255.mRNA.1	0
Lys	—	—	DIPPA_07350.mRNA.1,2	0
Met	DIPPA_09572.mRNA.1	0.669	DIPPA_18776.mRNA.1	0
Phe	—	—	DIPPA_20594.mRNA.1	0
Pro	—	—	DIPPA_06165.mRNA.1	0
Ser	—	—	DIPPA_04467.mRNA.1	0
Thr	—	—	DIPPA_01624.mRNA.1	0
Trp	DIPPA_01574.mRNA.1	0.745	DIPPA_08432.mRNA.1	0
Tyr	—	—	DIPPA_28314.mRNA.1	0
Val	—	—	DIPPA_28163.mRNA.1	0

^a^ For localisation prediction and weighted average (WA) scores, see Methods

A recent proteomic study utilizing affinity pull-down of mitoribosomal complexes has revealed miniature rRNAs embedded in an exceptionally protein-rich mitoribosome, the assembly of which is apparently mediated by an outsized cohort of cofactors [14]. At 5 MDa, the *Diplonema* mitoribosome contains as many as 130 integral mitochondrial ribosomal proteins (mt-RPs) and has a protein: RNA ratio of 11:1. The protein composition (listed in category D, Supplementary Table S2) reflects a mixture of (1) conserved bacteria-like mt-RPs contributed by the α-proteobacteria-like ancestor of mitochondria; (2) subsequently-introduced eukaryote-specific mt-RPs tracing their evolutionary origin back to the last eukaryotic common ancestor (LECA); and (3) homologs of novel mt-RPs first found in the kinetoplastid mitoribosome, supplemented by (4) some three dozen newly identified diplonemid-specific mt-RPs. In addition, this study identified > 50 candidate assembly factors, around half of which contribute to early mitoribosome maturation steps. Several other conserved ribosome assembly factors not recovered in the affinity pull-down experiments were identified here, including three copies of a protein homologous to MAM33, an evolutionarily conserved mitochondrial matrix protein that is proposed to bind specific mt-RPs to ensure proper assembly [67]. This inventory of mt-RPs and proteins involved in mitoribosome assembly greatly exceeds what has been described in other mitochondrial systems.

We retrieved a mixture of RNA modification enzymes, some specific for tRNAs, others for rRNAs and some with dual specificity, mostly methylases and pseudouridine synthases. Because all tRNAs functioning in the *Diplonema* mitochondrion are presumed to be cytosolic-type and imported into the organelle (by the mitochondrial tRNA import protein MTR, also identified in the mitoproteome), mitochondrial tRNA modification enzymes may function to re-tailor imported tRNAs to increase their compatibility with the mitochondrial translation system, as has been demonstrated in kinetoplastids [68, 69]. In *T. brucei* and *Leishmania tarentolae* mitochondria, cytidine deaminase is responsible for a specific C to U modification in the first position of the anticodon allowing the imported tRNA^Trp^ to decode mitochondrial UGA codons as tryptophan [58, 70]. We assume a parallel modification of imported tRNA^Trp^ must occur in *Diplonema* mitochondria and have identified a cytidine deaminase (DIPPA_33495) as a likely homolog of this enzyme, termed TbmCDAT in *T. brucei*.

### Membrane

General membrane proteins identified here include three prohibitins, as well as transmembrane proteins 53-like, 14 C-like, two 65-like and two Mpv17/PMP22 family members. All of these membrane proteins have a high mitochondrial localization score (WA score > 0.65). We also identified two paralogs of the apoptosis-related protein Bax1 inhibitor.

We did not retrieve homologs of Mmm1, Mmm2/Mdm34, Mdm10, and Mdm12, subunits of the outer membrane endoplasmic reticulum-mitochondria encounter structure (ERMES), which usually mediates associations between mitochondria and the endoplasmic reticulum [71]. We did, however, find a homolog of the ERMES protein Gem1 (MIRO mitochondrial rho GTPase) [72]; in the absence of evidence of a conventional ERMES complex in *Diplonema*, the role of this protein is unclear. Similarly, our searches failed to find any evidence of conventional subunits [73] of the mitochondrial contact site and cristae-organizing system (MICOS), which directs the formation of cristae [74]. A diverged and expanded MICOS complex has been characterized in *T. brucei* [75], and in *Diplonema* we did identify three of the nine subunits of this complex (Mic20, Mic34 and Sam50), although Mic20 and Mic34 candidates are considerably divergent compared to their kinetoplastid counterparts. While our results in this regard are inconclusive and will have to be augmented by additional, likely proteomics, analyses, they do point to diplonemids having a kinetoplastid-type MICOS.

Few mitochondrion-targeted proteins involved in mitochondrial fusion and fission were confidently identified in our study: a homolog of mitochondrial fission process protein MTFP1 and two dynamin-like proteins (DLPs), one of which (DIPPA_20507.mRNA.1) is a homolog of newly characterized *Tb*MfnL in *T. brucei*. *Tb*MfnL is a DLP anchored to the inner mitochondrial membrane and part of a novel membrane remodeling system [76]. Two other closely-related *D. papillatum* DLPs are not predicted to be mitochondrial (WA scores = 0) but are included in our list because they are evident orthologs of a single *T. brucei* DLP implicated in mitochondrial division [77–79].

**Fig. 2:**
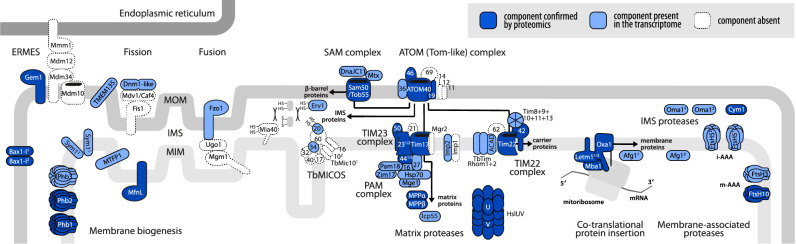
Schematic view of selected components and pathways involved in organelle biogenesis and protein trafficking and turnover in the *D. papillatum* mitochondrion

Fusion/fission proteins identified in the *Andalucia* mitoproteome but apparently missing from *Diplonema* (as well as from kinetoplastids) include homologs of bacterial cell division proteins FtsZ1 and FtsZ2 and septum site-determining proteins MinC, MinD and MinE. We did identify a homolog of transmembrane protein TMEM135, thought to regulate the balance between mitochondrial fusion and fission. This protein has been described in fungi and animals and is also present in *Andalucia* [17]. The prominent components involved in membrane biogenesis are illustrated in Fig. 2.

Arrows indicate protein trafficking pathways. Dark blue shades highlight assigned components whose presence was confirmed using mass spectrometry, while light blue shades indicate those identified in the transcriptome and genome sequence data. Absence of a clear homolog, when of particular significance, is depicted in white. Membranes and other features of the mitochondrion are shown in grey shades. MIM, mitochondrial inner membrane; IMS, intermembrane space; MOM, mitochondrial outer membrane. Note that conventional MICOS, FtsZ-MinCDE division system and ClpXP protease complex are completely absent and thus not depicted.

### Protein and metabolite trafficking

#### Protein trafficking

##### Presequence (Classical) pathway

The canonical translocase of the outer membrane (TOM) comprises receptor proteins Tom20, Tom22 and Tom70, the channel-forming subunit Tom40 and three small proteins (Tom5, Tom6 and Tom7) [80]. *Diplonema* appears to lack a canonical TOM complex, as we failed to find homologs of Tom40, Tom22, Tom6 or Tom7 (Fig. 2). We did, however, identify homologs of five of nine subunits of the atypical translocase of the outer membrane (ATOM) complex characterized in trypanosomatids [81, 82]: ATOM46, ATOM40 (~ Tom40) and ATOM19 (~ Tom5), as well as a highly diverged pATOM36 (peripheral receptor) and a putative Tom34 subunit. Other ATOM subunits reported to be present in *E. gracilis* [22], including ATOM11, ATOM12, ATOM14 and ATOM69, were not retrieved here. Failure to identify a *Diplonema* ATOM69 homolog is puzzling given that a *Euglena* counterpart was readily picked up by BLASTp search. The apparent absence of ATOM69 is particularly noteworthy because in trypanosomes it displays a preference for presequence-containing substrates. As in *Euglena*, a homolog of ATOM14/Tom22, the single subunit conserved between opisthokonts and kinetoplastids, could not be found in *Diplonema*.

We identified three of five subunits of the TIM23 presequence translocase of the inner membrane: the core translocase subunits Tim23 (two paralogs) and Tim17, as well as Tim50; conversely, we did not find Tim21 or Mgr2/Romo1. Tim17, Tim23 and Tim50 constitute the membrane-anchoring component, with Tim50 traditionally functioning as a receptor for precursors, whereas Tim17 and Tim23 serve as the pore-forming units [82].

All expected components of a canonical presequence translocase-assisted motor (PAM) complex are present in *Diplonema*, as they are also in *Euglena* [22]: Hsp70 (DnaK), Tim44 (two distinct versions), Tim14/Pam18 (two paralogs), Tim16/Pam16, Mge1 (co-chaperone GrpE) and chaperone Zim17 (Hep1). Curiously, in *Trypanosoma*, Pam18 and Pam16 orthologs do not participate in protein import, but rather have been repurposed to regulate mtDNA replication [83]. In *T. brucei*, these two proteins have been functionally replaced by a new component, Pam27 [84]; *Diplonema*, as well as other diplonemids, encode a homolog of this protein (DIPPA_31051), raising the possibility that kinetoplastid and diplonemid PAM complexes might share this derived protein import feature as well. In *Diplonema*, as in *Euglena*, only one subunit (Imp2) of the inner membrane peptidase complex was retrieved, with Imp1 not detected. Proteins involved in matrix presequence processing are present: α and β subunits of a matrix processing peptidase, Icp55 (intermediate cleaving peptidase 55), three distinct intermediate peptidases, two M3 family metallopeptidases and a Cym1/Mop112 homolog (pitrilysin family M16 presequence metallopeptidase).

We identified Oxa1, core chaperone of the oxidase assembly (OXA) complex, which facilitates insertion of proteins synthesized by mitoribosomes into the mitochondrial inner membrane. In yeast, paralogous Mdm38 and Ylh47 proteins, having overlapping functions, serve as ribosome receptors, assisting in co-translational protein insertion [85]. In *Diplonema*, we found two variant LETM1/MDM38 family proteins, DIPPA_07548 and DIPPA_01289, that presumably fulfill this role, although based on phylogenetic trees, it seems that the two diplonemid paralogs are not as recent as the yeast Mdm38 and Ylh47. Moreover, DIPPA_07548 is more similar to the single *E. gracilis* MDM38/LETM1 and to some protist counterparts, whereas DIPPA_01289 is closer to the single kinetoplastid protein, suggesting that the two *Diplonema* proteins might specialize in their activities. A third OXA component, Mba1 [86], is considered to be the fungal equivalent of mammalian mitoribosomal protein L45 [87, 88]. While kinetoplastids have an extremely divergent Mba1-like protein [89], it is not a mitoribosomal component but is instead involved in ETC biogenesis. Similarly, *Diplonema* lacks L45 [14], but contains the Mba1-like homolog (DIPPA_23756; category F). Nor did we find a clear homolog of Mrx15/TMEM223, another ribosome receptor that in yeast, together with Mba1, organizes co-translational insertion and protein biogenesis in mitochondria [90]. Both Mba1/L45 and Mrx15/TMEM223 are present in the *Andalucia* mitoproteome [17].

##### Carrier pathway

*Diplonema* has five proteins that are homologs of trypanosomatid small Tim proteins: Tim8/13, Tim9, Tim10, Tim11 and Tim 13. These proteins are intermembrane space chaperones that aid in the import of mitochondrial inner membrane proteins and are essential for biogenesis of the single translocase of the inner membrane (TIM) complex [91]. All except Tim13 contain a zf-Tim10_DDP (PF02953) domain and all display a twin Cx_3_C motif. A homolog of trypanosomatid Tim12 (Tbr927.4.3430) was not retrieved.

In trypanosomes, a single translocase of the inner membrane (TIM) complex appears to substitute for separate conventional TIM22 and TIM23 complexes [81, 82]; however, as noted above, a canonical TIM23 complex is present in *Diplonema* as well as in *Euglena* [22]. The single trypanosomatid TIM comprises six subunits [92]: TbTim17 (ortholog of canonical Tim22), TbTim42, TbTim62, TbRhom I, TbRhom II and ACAD (acyl-CoA dehydrogenase). *Diplonema* encodes homologs of all of these proteins except TbTim62. Notably, *Diplonema* has homologs of the rhomboid-like novel trypanosomatid proteins TimRhom I and II, which appear to be absent in *Euglena.*

The mitochondrial sorting and assembly machinery (SAM), also known as the TOB complex, mediates the topogenesis of precursors of beta-barrel proteins, which in turn, mediate communication between cytosol and mitochondria. We identified Tob55/Sam50, present in kinetoplastids and *Euglena* (the latter has two genes, the protein sequences of which both retrieved the lone *Diplonema* Tob55/Sam50), along with a single putative metaxin-like protein.

The canonical mitochondrial intermembrane space import and assembly (MIA) machinery consists of two proteins, Mia40 (an oxidoreductase) and Erv1 (a sulfhydryl reductase), operating together as a disulfide relay. We identified in *Diplonema* Erv1 but not Mia40, which seems to be absent from euglenozoans, including *Euglena* [22], as well as a number of other protist lineages [93]. From phylogenetic considerations, the latter authors suggested that the ancestral MIA pathway required only Erv1, with O_2_ likely substituting for Mia40 as the physiological oxidant for Erv1, although in trypanosomes this protein seems to be involved in several mitochondrial functions [94]. Mia40 has been reported in *Andalucia* [17], which argues that this protein was, in fact, present in the discobid common ancestor, and perhaps also in the LECA. The twin Cx_9_C proteins listed in Table 2 must presumably pass through a MIA-like system during import [95]. Therefore, it is unlikely that Mia40 or a Mia40-like activity is truly absent in *Diplonema* mitochondria; at the moment, however, this issue remains unresolved.

We found no evidence of the bacteria-like twin-arginine translocation (Tat) [96] pathway or the recently reported mitochondrial type II secretion system (T2SS) [97], both present in *Andalucia* [17]. The Tat pathway transports folded proteins across the cytoplasmic membrane of most bacteria and archaea, but whether any *Diplonema* mitochondrion-targeted proteins are imported as fully folded proteins is unknown.

#### Metabolite trafficking

##### Mitochondrial Carrier (MC) System 

The *Diplonema* mitoproteome contains a rich inventory of mitochondrial carrier (MC) proteins (SLC25 family) mediating the transport of metabolites from the cytosol into mitochondria. We identified 66 proteins having at least one MC_carr (PF00153.27) domain and falling into 19 KEGG classes (Table 4). An additional nine members lack KEGG annotation. Two-thirds of the classes have multiple members, with the KEGG orthologs K05863 (adenine nucleotide; 12 members) and K15109 (carnitine/acylcarnitine; nine members) especially prominent. The number of *Diplonema* MC proteins approximates or even exceeds the number (>50) found in mammalian [98] and plant [99] mitochondria.

**Table 4:** Mitochondrial carrier (MC) proteins in *D. papillatum*^a^

KO Entry^b^	KEGG Member^b^	No.	Predicted Substrate Specificity
K05863	4/5/6/31	12	adenine nucleotide
—	—	1	ADP/ATP
K13577	10	1	dicarboxylate
K14684	23/24/25/41	4	phosphate
K15085	42	2	—
K15100	1	2	citrate
K15102	3	3	phosphate
K15103	8/9	2	uncoupling protein
K15104	11	3	oxoglutarate
K15105	12/13	2	aspartate/glutamate
K15106	14/30	2	—
K15109	20/29	9	carnitine/acylcarnitine
K15110	21	4	2-oxodicarboxylate
K15111	26	3	S-adenosylmethionine
K15112	27	2	uncoupling protein
K15113	28/37	1	iron
K15115	32	1	folate
K15116	33/36	1	—
K15117	34/35	1	—
K15119	39/40	1	—

^a^ Proteins having one or more MC_carr (PF00153.27) domains

^b^ See Supplementary File S2, (F) Protein & Metabolite Trafficking, for sequences of individual MC proteins, which were annotated via the KEGG Automatic Annotation Service (KAAS) using default parameters. Entries not assigned a specific KAAS number are nevertheless predicted to be solute carrier family 25 (SLC25) members

##### Other Non-MC Transporters

Surprisingly, a voltage-dependent ion channel (VDAC; porin), responsible for the transport of metabolites and ions across the outer mitochondrial membrane, appears to be absent in *Diplonema*, although it is present in trypanosomatids [100]. A porin-like protein has been reported for *Euglena* (AAG38111.1); however, this and a variety of other porin queries failed to retrieve a significant BLASTp hit from *Diplonema*, although we did find two porin-like proteins, DIPPA_08220 and DIPPA_08223. In the absence of porin, ions may be transported via specific channels. For example, a calcium uptake system [101] is present in *Diplonema*: we retrieved two alternatively spliced isoforms of a mitochondrial calcium uniporter (MCU) as well as three additional MCU domain-containing proteins. Three calcium-binding, EF-hand superfamily proteins were also found, one of which (DIPPA_17095) appears to be the ortholog of human calcium uptake protein MICU1. We identified additional non-MC enzymes, including tricarboxylate/iron (sideroflexin; two variants), pyruvate, and divalent metal cation (Fe/Co/Zn/Cd) transporters.

### General metabolism

The reticulated mitochondrion of *Diplonema* is predicted to engage in a complex metabolism that includes numerous pathways that are ubiquitous among conventional mitochondria throughout eukaryotes. These pathways include iron-sulfur cluster biosynthesis (ISC assembly, ISC export, Fe^2+^ import), glycine cleavage, branched chain amino acid degradation, porphyrin (heme) biosynthesis, ubiquinone biosynthesis, fatty acid oxidation (FAO) and cardiolipin metabolism.

Two distinct variants exist for several ISC components, including IscA, Yah1, Nfu1 and BolA. In contrast to *Andalucia*, which appears to lack the regulatory component of the glycine cleavage system [17], *Diplonema* has two mitochondrion-targeted variants. In the ubiquinone biosynthesis pathway, we identified two non-paralogous, atypical ABC1 (activator of bc1 complex) kinase family proteins as Coq8 candidates, as well as two variant Coq10 homologs. We did not recover clear Coq6 or Coq7 homologs nor a Coq9 homolog, even though we were successful in retrieving Coq9 candidates from kinetoplastid and *E. gracilis* proteomic data. We recovered a putative candidate Coq11, functionally characterized in *S. cerevisiae* [102] and *Schizosaccharomyces pombe* [103], but did not identify Coq12.

Cardiolipin is an important component of the mitochondrial inner membrane. The cardiolipin metabolism pathway of *Diplonema* is puzzling. Whereas in eukaryotes this pathway is localized exclusively in mitochondria [104], and predicted to be so also in *Andalucia* [17], our analysis indicates that most of the enzymes involved are non-mitochondrial in *Diplonema*. Moreover, we failed to identify the first enzyme of the pathway, the mitochondrial matrix protein Mmp37/Tam41 (CDP-DAG synthase), which also appears to be absent in euglenids; while the gene is retained in kinetoplastids [105], the corresponding protein is non-mitochondrial. *Diplonema* homologs of Ups1, Ups2 and Ups3, related small proteins that control phospholipid metabolism in the mitochondrial intermembrane space (IMS), were also not identified. Ups protein import is mediated by Mdm35p [106], which we did in fact identify. Considering these peculiarities, further investigation of cardiolipin biosynthesis throughout Euglenozoa is warranted.

In addition to harboring the canonical mitochondrial ISC and nucleo-cytosolic CIA systems for iron-sulfur cluster biosynthesis, *Diplonema* encodes components of a SUF system, which is normally found in bacteria and plastids [107] but also in some anaerobic protists [108]. We identified two subunits, SufD (DIPPA_35210) and SufE (DIPPA_10557) with predicted targeting to mitochondria, although their WA scores are just at the cut-off (0.491 and 0.502, respectively). Three other subunits—SufB (DIPPA_05262), SufC (DIPPA_19968) and SufS (DIPPA_09353)—appear to be non-mitochondrial. Since we conclude that the SUF system is likely cytosolic in *Diplonema*, the SufD and SufE proteins are not included in our list of candidate mitoproteins. An apparently homologous SUF system has been reported in *Euglena*, localized to its plastid [109, 110], but has evidently been lost in kinetoplastids.

In addition to proteins of the specific pathways mentioned above, we identified numerous oxidoreductases (76), transferases (78), hydrolases (47), lyases (21), isomerases (15) and ligases (9) participating in a wide range of other pathways, including synthesis and degradation of amino acids, nucleotides, fatty acids, cholesterol, coenzymes and one-carbon fragments. Although in many cases the specific pathway(s) and functional role(s) of these proteins is/are evident, in most instances their precise contribution to mitochondrial metabolism in *Diplonema* remains to be elucidated.

### Protein folding, processing and degradation

Among molecular chaperones, we retrieved homologs of two Hsp10, an Hsp33, three Hsp60, five Hsp70 (including four paralogs encoded on the same genomic scaffold) and an Hsp75. All of these entries are strongly predicted to be targeted to mitochondria (WA scores > 0.8). Notable in this category is an unusually large number (31) of mitochondrion-targeted DnaJ domain-containing proteins. The possible involvement of this set of proteins in mitochondrial RNA processing, likely in the formation and stabilization of ligation and/or editing complexes, is discussed below. We also identified two copies of the ATP-binding subunit of Clp protease, as well as two proteins that regulate/modulate chaperone activity: a BAG domain-containing protein and co-chaperone YbbN.

Various conserved proteins known to be involved in mitochondrial protein processing and degradation were identified, including two AAA+ proteases, m-AAA+ and i-AAA+, whose catalytic sites are on opposite surfaces of the MIM (facing the matrix and IMS, respectively) and function in the selective degradation of misfolded and excess polypeptides [111]. A second ATP-dependent protease, HslVU, is also present in the *Diplonema* mitoproteome, with three variants of subunit HslU (ClpY) and one HslV (ClpQ) subunit retrieved (Fig. 2). Other proteases/peptidases identified are two copies of an ATP-dependent zinc metallopeptidase FtsH; a rhomboid family intramembrane serine protease; a peptidase S9 family protein (prolyl oligopeptidase); an Oma1 zinc metallopeptidase-like protein; and a M48 family metallopeptidase.

As in *Trypanosoma*, we recovered two copies of AFG1/ZapE, a protease-associated ATPase localized to the matrix side of the MIM. Its kinetoplastid homolog is involved in regulating respiratory complexes *via* its Oxa1 interaction [112] and its mammalian homolog, LACE1, was shown to mediate turnover of nucleus-encoded CIV subunits [113].

### Reactive oxygen species (ROS) metabolism, regulation

Reactive oxygen species (ROS) are produced in the eukaryotic cell by beta-oxidation of fatty acids, oxidation of proteins, and mitochondrial electron transport. Accordingly, detoxification of ROS takes place in the peroxisomes, cytosol, and mitochondria. Identified *Diplonema* mitoproteins involved in the ROS metabolism are exceptionally numerous and include a homolog of peroxiredoxin, which plays a major role in metabolizing hydrogen peroxide in the organellar matrix [114]. Other ROS-protective proteins recovered include five thioredoxin domain-containing proteins, two glutaredoxins, two cytochrome *c* peroxidases (Ccp1), two heme-dependent peroxidases and a superoxide dismutase. We also found a mitochondrial peptide-methionine (R)-S-oxide reductase, which plays an important role in the antioxidant response by reducing the S-stereoisomer of methionine sulfoxide (MetSO) to methionine.

Possible protein-modifying regulatory enzymes present in the analyzed mitoproteome include six serine/threonine-protein phosphatases and seven histidine phosphatases. An additional three proteins are ABC1 atypical kinases, probable serine/threonine-protein kinases that contain an AarF domain (COG0661), implicated in the regulation of ubiquinone biosynthesis. Lastly, we identified a single SIR2 family protein (NAD-dependent protein deacetylase) and two proteins containing an RCC1 (regulation of chromosome condensation) domain.

### Unknown function

Fully one-third of the candidate mitoproteins (691) in *D. papillatum* could not be characterized as to specific function and/or enzymatic activity. Importantly, the proteins in this category (J) that we could detect by mass spectrometry in proteomics experiments are not outliers but have abundances in line with functionally assigned proteins (Fig. 1B). While the rate of detection (as a percentage of proteins in a given category) is quite low for category J proteins (9%), it is similar to that of category C proteins (DNA and RNA Metabolism; 10%) (Fig. 1 C, D).

About 9% of these unknown function proteins (Class 1) contain one or more putative conserved domains, which in many cases provide an indication of their general function. For example, SET domain-containing proteins, four of which are listed in Class 1, have protein lysine methyltransferase activity [115]. No clearly identifiable domain(s) is/are present in another subset (~ 7.5%) of these uncharacterized mitoproteins, which are nevertheless conserved in sequence (Class 2). About one-third of Class 2 proteins are euglenozoan-specific, shared exclusively with kinetoplastids or with kinetoplastids and *E. gracilis*. Another one-third of Class 2 is more widely distributed among eukaryotes, but usually with a punctuate distribution. A final third of Class 2 proteins have bacterial homologs (usually in Pseudomonadati but also Bacillati) as their top hits. Notably, proteins in Class 2 appear to be almost entirely absent from jakobids and malawimonads.

The majority (~ 84%; 579 entries) of the unknown function category comprises proteins that have no conserved domains and no evident homologs outside diplonemids (Class 3). Importantly, the vast majority of these proteins are expressed under regular cultivation conditions (> 95% detected at the transcript level and ~ 10% at the protein level as well). To assess the distribution of Class 3 proteins within diplonemids, we carried out a BLASTp survey using *D. papillatum* sequences as queries against 10 additional diplonemid species for which we have predicted proteome data. We binned the results as follows: Subclass 3.1 (17%), BLASTp hits in all diplonemids; Subclass 3.2 (51%), BLASTp hits in one to nine but not all 10 diplonemids; Subclass 3.3 (32%), no BLASTp hits outside of *D. papillatum*. Thus, 20% of the inferred *D. papillatum* mitoproteome (393 proteins) consists of diplonemid-specific proteins of unknown function, with a further 10% (186 proteins) seemingly exclusive to this species.

A particularly interesting sub-group within the unknown function category is composed of proteins having a twin Cx_9_C (CHCH) structure. Four proteins of this type are in the conserved unknown group, whereas five others are in the diplonemid-specific group (Table 2). As mentioned earlier, a twin Cx_9_C motif characterizes certain ETC subunits and CIV assembly proteins. Accordingly, it is possible that some of these unknown proteins represent either unrecognized, highly diverged versions of ‘missing’ conventional ETC subunits or assembly factors; novel ETC subunits, as in the case of CI NDUDP1 [27]; or novel assembly factors. The MIA protein Mia40, which we failed to find in *Diplonema*, is another twin Cx_9_C motif protein, but its homolog is not among the unknown mitoproteins.

One Cx_9_C motif protein (DIPPA_22896), 72 amino acids long, has an especially interesting taxonomic distribution: seemingly ubiquitous among protists but selectively absent from jakobids and malawimonads, and present in Fungi but absent from Metazoa and Viridiplantae. A protein exhibiting such a patchy distribution qualifies as a jötnarlog: defined as “a protein found in sufficiently diverse eukaryotic taxonomic supergroups to infer a common origin concurrent with or pre-dating the LECA, but hidden from previous cell biological investigations due to loss or divergence in yeast and animal model systems” [116]. This class of proteins, belonging to different cell compartments, was recently identified as particularly abundant in diplonemids [117].

By virtue of sequence conservation in the absence of known function, the Class 2 group of *Diplonema* mitoproteins deserves further consideration and investigation. Sequence conservation usually reflects an evolutionary ancestral role, and the broader the phylogenetic distribution, the more fundamental that role is likely to be to mitochondrial structure and/or function.

### Unusual transcript processing in diplonemid mitochondria

#### Bioassembly of mitochondrial transcripts

As noted above, diplonemids have unprecedented mitochondrial gene structure, whereby genes are systematically broken into pieces, with up to 11 per gene in *D. papillatum* [9, 56, 118, 119] and up to 24 per gene in *Namystynia karyoxenos* [12]. These gene fragments, referred to as modules, are encoded individually on multiple circular chromosomes (80 in the type species) and are transcribed independently into module transcripts. Mature mRNAs and rRNAs are generated by joining module transcripts in the correct order. The bonding process has been referred to as ‘*trans*-splicing’; however, in the absence of introns and exons, this process is better described as ‘transcript ligation’. How transcript assembly in diplonemids works is as yet mysterious, with two main issues unresolved: (i) the catalytic activity that concatenates module transcripts and (ii) the accurate selection (matching) of cognate partners.

The gene module transcripts in diplonemids are covalently joined together. While a ribozyme-based mechanism could theoretically catalyze this step, the lack of conserved nucleotides at the module junctions that are characteristic for ribozymes suggests otherwise [120]. This finding leads us to conclude that ligation is achieved by a proteinaceous RNA ligase, likely a member of the RtcB family [121, 122]. This inference comes from the fact that this specific enzyme class is the only one known to be capable of ligating the atypical termini of *Diplonema* module transcripts, which consist of 3ʹ phosphate and 5ʹ hydroxyl groups [10].

Genes encoding RtcB-like proteins have been found in diverse protist groups and metazoans, but are mostly absent from fungi and land plants [123]. The genome of *D. papillatum* encodes three RtcB-like proteins [24], which are very likely functional because they possess all function-critical residues in the catalytic site (Supplementary Figure S1), and because homologs are present in most transcriptomes of other diplonemids investigated.

**Fig. 3:**
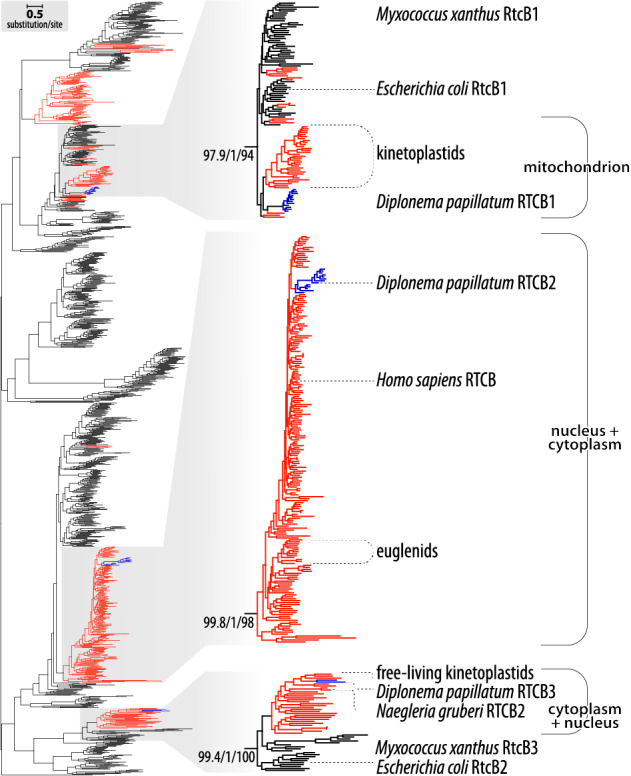
Diplonemid RtcB proteins form three distinct clades with different predicted subcellular localization. Phylogenetic tree of RtcB family proteins from diplonemids (blue), other eukaryotes (red) and prokaryotes (black). The collection contains 1,183 sequences, notably 662 from bacteria, 91 from archaea, 25 from viruses and the rest from eukaryotes, out of which protist sequences represent ~ 95%. The expanded views (right side) depict the three clades of interest with branch-support values (SH-aLRT support (%)/aBayes support/ultrafast bootstrap support (%)), and the proteins’ subcellular localizations predicted by DeepLoc2: most DpRTCB1-related proteins are mitochondrial, while DpRTCB2- and DpRTCB3-related proteins are nuclear and/or cytoplasmic

In a phylogenetic analysis encompassing RtcB sequences from all domains of life, the diplonemid proteins RTCB1, 2 and 3 each associate with distinct clades (Fig. 3) mostly composed of bacterial counterparts. This grouping pattern indicates that the three diplonemid proteins are not paralogs that arose by gene duplication in a common eukaryotic ancestor, but are rather independent acquisitions of bacterial RTCB genes *via* horizontal gene transfer. Of note, one of these putative ligases, RTCB2 (DIPPA_32518), which is predicted to reside in the nucleus, is part of a clade that includes tRNA-splicing ligases from Archaea and Metazoa [124, 125]. We therefore consider RTCB2 to be a tRNA ligase homolog, which is corroborated by the presence of nucleus-encoded intron-containing tRNA genes in *D. papillatum*.

In the type species, only one of these proteins, DIPPA_22497, referred to as RTCB1, is of predicted mitochondrial localization and is therefore the prime candidate for catalyzing the module transcript ligation in mitochondria. RTCB1 associates with clade 1 (Fig. 3), a group that contains just a few additional eukaryotes, notably *Naegleria* and kinetoplastids. Only *Trypanosoma* RtcB has been investigated and was shown to be incorporated into the mitochondrial matrix [126] (but see also [127]), although its precise function remains unclear [128]. Otherwise, clade 1 consists predominantly of bacterial members, among which are the experimentally well-examined proteins from *Myxococcus xanthus* (MxRtcB1) and *E. coli* (EcRtcB1) [129–131]. Whereas the *E. coli* protein was reported to repair 16 S rRNA cleaved by endogenous ribotoxins produced in response to cell stress [132, 133], it is more efficient in re-ligating tRNAs cleaved by colicin E3 [134], a secreted endoribonuclease implicated in bacterial warfare. It appears therefore that the principal biological role of EcRtcB1 lies in the remediation of ribotoxin-induced damage inflicted by competing bacteria.

The *Naegleria*, kinetoplastid and diplonemid RtcB family sequences within clade 1 likely diversified from a shared predecessor acquired by their last common ancestor. We postulate that over evolutionary time, the gene has changed substrate specificity to accommodate various functions, including the joining of mitochondrial module transcripts in diplonemids.

Beyond catalytic activity, the second unresolved question is how match-making of cognate transcript modules is assured, prior to their assemblage. As the modules lack reverse-complementary sequence elements that would allow them to align through base pairing, match-making is likely achieved by *trans*-acting factors [10]. The involvement of *trans*-factor RNAs is unlikely, given that antisense RNAs were not detected for a large number of experimentally tested *D. papillatum* junctions [10]. Consequently, the ~ 65 distinct junctions within the *D. papillatum* transcriptome are probably aligned by RNA-binding proteins (RBPs). The potential candidates for this role are discussed below.

#### Mitochondrial RNA editing

In numerous mitochondrial systems, RNA editing is crucial for producing functional mRNAs [135]. Diplonemid mitochondria are notable for also undergoing otherwise rare rRNA editing [13, 14]. In these protists, two distinct types of mitochondrial RNA editing were observed. One involves the addition of up to ~ 50 uridylate residues (Us) to the 3ʹ end of module transcripts prior to their assemblage [119]. When the module in question is internal, as in the cytochrome *c* oxidase subunit *cox1*, these appended Us appear as insertions within the mature transcript [120].

The second type of diplonemid mitochondrial RNA editing involves nucleotide substitutions, replacing not only C by U, but also A by I (inosine) [11]; the latter editing has so far not been described in other mitochondrial systems. Additional, unusual types of RNA editing were observed in the hemistasiid clade of diplonemids, including apparent A-appendage and G-to-A substitutions [12].

#### Candidates for RNA editing enzymes

The presence of non-genome-encoded U appendages at the 3′-termini of module transcripts suggests the involvement of a terminal nucleotidyltransferase. To identify potential enzymes responsible for this activity, we compiled PFAM profile HMMs of domains commonly found in such enzymes. Domains included Ret2_MD (PF18528), PAP_assoc (PF03828) and terminal uridylyl transferase (TUTase) (PF19088), along with poly(A) polymerase domains as a negative control (Supplementarty Table S3 of Supplementary File S1). The search within the *D. papillatum* mitoproteome using these profile HMMs revealed two potential TUTases. DIPPA_04001 yielded the strongest hit among all PFAM models with PAP_assoc (E-value 2e-09). The absence of a significant match with the TUTase domain is not surprising because due to the bias of PF19088 toward animal sequences, this profile HMM does not even retrieve functionally confirmed trypanosomatid TUTases.

The second TUTase candidate is DIPPA_34584. It is the only mitoprotein that exhibited significant albeit weaker matches with both the PAP_assoc and TUTase domains (E-value ~ 7e-03). Phylogenetic analyses (not shown), which included confirmed eukaryotic TUTases and mitochondrial poly(A) polymerases, indicated a slightly stronger affiliation of DIPPA_04001 with TUTases, and DIPPA_34584 with poly (A) polymerases. Based on these findings, we consider both DIPPA_04001 and DIPPA_34584 to be the primary candidates responsible for catalyzing the U-appendage RNA editing in *Diplonema* mitochondria.

The hypothesis that DIPPA_04001 functions as a mitochondrial RNA editing enzyme is further supported by the fact that trypanosomatid TUTases RET1 and RET2, which are involved in mitochondrial U insertion RNA editing and uridylylation of guide RNAs, also carry the PAP_assoc domain [136]. In addition, DIPPA_04001 possesses all TUTase-specific residues in its catalytic site (Supplementary Figure S2) and its predicted three-dimensional structure aligns closely with the crystal structure of RET1 [137] and RET2 [138]. Notable differences, both in structure and sequence, are confined to the central regions of the trypanosomatid and diplonemid proteins. These differences are expected because the middle region of the trypanosomatid enzymes has been shown to be involved in the binding of protein partners [139] or their substrate [140].

**Fig. 4:**
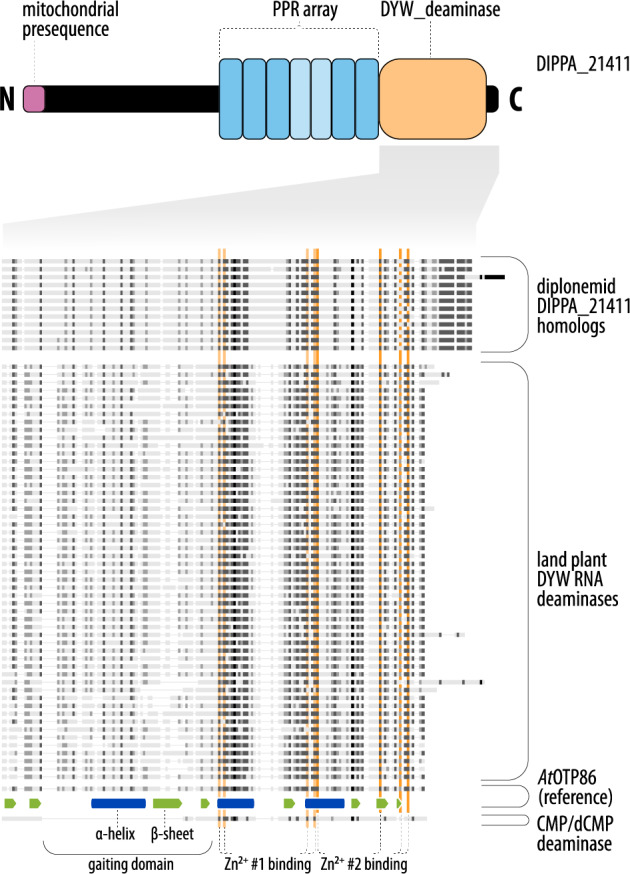
Diplonemid proteins of the DYW_deaminase family carry a PPR-motif array and Zn-ion binding sites. *Top section*, the domain arrangement of the DYW deaminase from *D. papillatum* (DIPPA_21411), a domain profile conserved across its diplonemid homologs. N, amino terminus; C, carboxy terminus. In diplonemids, the PPR array, typical for DYW deaminases from land plants, comprises five motifs of 35-residue length (blue) and two motifs of 31 residues (light blue). *Middle section*, the DYW_deaminase-like domain (orange) and C-terminal portion of diplonemid proteins, corresponding to amino acids 825–960 of the reference protein OTP86 from *Arabidopsis thaliana*, were aligned with those from plant counterparts. Grey shades indicate the degree of sequence similarity with dark and light corresponding to higher and lower conservation, respectively. For more details, see Supplementary Figure S3. *At the bottom*, the structural regions of the reference are depicted in green and blue bars, and functional domains are annotated, including the proposed regulatory interface (gating domain), and residues involved in the binding of two zinc ions (Zn^2+^; light and dark orange bars). The portion of diplonemid proteins aligning with the gating domain of the reference protein is less well conserved than that corresponding to the catalytic core. Note that the deaminases that use free CMP/dCMP as substrate (e.g., *E. coli* Cdd) lack the gating domain, as well as the second Zn ion-binding site

The second type of RNA editing in diplonemid mitochondria is C-to-U and A-to-I substitutions *via* base deamination, demonstrated experimentally in *D. papillatum* [11, 119]. Therefore, we searched the *D. papillatum* proteome for conserved domains typically found in plant proteins that mediate site-specific deamination in organellar pre-mRNAs. These domains include pentatricopeptide repeat (PPR) (PF01535 and PF13041) and DYW_deaminase (PF14432.9) domains [141–143]. The search with these PFAM models retrieved DIPPA_21441, which matches all three domains (Fig. 4; Supplementary Figure S3). Since several deaminases from other organisms [144] and those evolved in vitro [145] are capable, albeit with low efficiency, of deaminating both Cs and As, we consider DIPPA_21441 a promising candidate for catalyzing C-to-U as well as A-to-I substitution RNA editing in *Diplonema* mitochondria.

### How are RNA-editing sites recognized?

Unlike the U-insetion/deletion type of RNA editing in kinetoplastid mitochondria, which involves guide RNAs [146], U-additions in *Diplonema* do not appear to be directed by such RNAs, as single-stranded molecules of 50–70 nt in length and high abundance were not detected in this organism [10]. Moreover, there is no evidence of antisense RNAs complementary to the 3′ end of the edited module transcripts and the corresponding poly(U) tract in *D. papillatum* [11]. Supported by the lack of a common sequence motif near the 16 editing sites, we infer that U-appendage is likely guided by as-yet-unidentified protein *trans*-factors, as discussed in the following section.

In the model plant *Physcomitrella*, PPR-DYW proteins not only catalyze C deamination in transcripts, but also recognize target-sites *via* their N-terminal array of the PPR motifs. Most *Physcomitrella* editing sites are served by a dedicated member of the PPR-DYW protein family [147]. In contrast, the *D. papillatum* genome (and apparently those of the other diplonemids) encodes only a single PPR-DYW protein, the above-mentioned DIPPA_21441. Consequently, we posit that DIPPA_21441 primarily functions as a deaminase, while the recognition of actual editing targets is likely achieved by other, yet-to-be-identified proteinaceous specificity factors that interact with DIPPA_21441.

The exact number of such specificity *trans*-factors required for substitution RNA editing in *D. papillatum* remains uncertain. It is possible that each of the 114 distinct deamination sites necessitates a unique factor. Alternatively, one factor could be responsible for each of the six densely packed editing clusters, plus an additional factor for the stand-alone deamination site [11]. Regardless of the specific scenario, substitution RNA editing in diplonemid mitochondria likely involves complex and dynamic multi-subunit editosomes, similar to those observed in flowering plants [148].

#### Large nucleus-encoded mitoprotein families in *D. papillatum*

As argued in the previous sections, both transcript assemblage and RNA editing in diplonemid mitochondria are most probably facilitated by proteinaceous specificity *trans*-factors that belong to large RNA-binding protein (RBP) families. Therefore, we analyzed the predicted *D. papillatum* mitoproteome for large protein families using BLAST, HMM searches with whole-protein profile HMMs and, in the case of PPR proteins, searches with protein-motif profile HMMs.

With more than 100 PPR proteins, this family is the largest identified so far in the *D. papillatum* mitoproteome. PPR proteins have been most extensively studied in plant mitochondria and chloroplasts, where they play a role in substitution RNA editing [63]. They are also involved in post-transcriptional gene regulation, from intron splicing to translation initiation in organelles and the nucleus across eukaryotes [149]. The identification of PPR proteins studied herein is detailed in the Supplementary File S1.

The second largest family of the *D. papillatum* mitoproteome, counting 30 members (Supplementary Table S2, category H), is characterized by a DnaJ domain. This domain is the most important motif of Hsp40 proteins, which act together with Hsp70 in multiple processes. Some DnaJ domain-containing proteins have been shown to interact with RNAs [150, 151], including proteins residing in mitochondria [152]. The DnaJ domain itself is apparently not involved in RNA binding but rather in protein-protein interaction [152].

Further, the mitoproteome includes 11 predicted DExD/H-box RNA helicases (Supplementary Table S2, categories B and C), a family whose members are recognized for their important roles in RNA metabolism, in particular pre-mRNA processing. Organelle-imported DEAD/DExH helicases have been reported to be involved in organelle intron splicing [153, 154], and mitochondrially-located family members in trypanosomes play a role in site- and substrate-specificity in pre-mRNA editing [155].

Finally, proteins of mitochondrial localization containing cold-shock-domains (CSDs, also termed Y box) comprise at least seven members in *Diplonema* (Supplementary Table S2, category C). The CSD is one of the most ancient and well-conserved nucleic acid-binding domains, found in proteins throughout bacteria and eukaryotes [156]. CSD-containing proteins, extensively studied in bacteria, vertebrates and plants but also *Plasmodium* [157], function among other roles as RNA chaperones in transcription and translation. The first reported organellar member of this family is RNA Binding Protein 16 (KRBP16) in trypanosomes, which regulates both RNA editing and stability, with its CSD interacting with the poly(U) tail of gRNAs [158].

Because of its large size, the PPR family is the most promising candidate for orchestrating the ~ 65 joining steps required during transcript assemblage in *D. papillatum* mitochondria. In turn, DnaJ, DExD/H-box and CSD domain-containing mitoprotein families could participate in guiding RNA editing. Despite 114 deamination sites in *D. papillatum*, this type of RNA editing might only require seven specificity factors under the condition that a single protein directs the editing of all sites within each of the substitution clusters. Similarly, the 16 U-appendage addition sites might be guided by a small number of protein factors provided that some of the latter recognize more than one site.

### Strategies to validate postulated specificity factors mediating transcript joining and RNA editing

In conclusion, the four large families of RBPs in the *D. papillatum* mitoproteome – PPR proteins, DnaJ domain-containing proteins, DExD/H-box helicases and cold shock domain proteins – are prime candidates as specificity factors in mitochondrial transcript assembly and RNA editing. This hypothesis can be validated by two approaches. The first involves protein tagging and affinity purification followed by mass spectrometry of protein complexes containing the presumed enzymes catalyzing RNA editing (DYW deaminase, TUTases) or transcript assemblage (RtcB RNA ligase). Co-purification of RBP proteins together with these enzymes would strongly indicate the involvement of the former in these activities. These methodologies have been implemented in *D. papillatum*, and experiments along these lines are in progress.

Second, it would be insightful to examine the number of PPR proteins and DnaJ, DExD/H-box helicase, and cold shock domain proteins in the mitoproteomes of other diplonemids. This analysis would allow us to determine whether the sizes of these protein families correlate, as in *D. papillatum*, with the number of RNA editing sites and transcript junctions. Such an investigation will be feasible once comprehensive mitoproteome data from the 10+ other diplonemids under study becomes available.

## Discussion

The predicted mitoproteome of *D. papillatum* comprises a total of 1878 proteins, one third of which were confirmed by mass spectrometry. Despite the extremely atypical structure of the *D. papillatum* mitochondrial genome and the equally bizarre organization and mode of expression of the genes it encodes, the predicted mitoproteome in this protist exhibits the essential hallmarks of a conventional mitochondrion. Among the basic functions ubiquitously distributed and conserved in aerobic mitochondria, we found in *Diplonema* energy conversion *via* the TCA cycle, ETC and oxidative phosphorylation; replication, transcription and translation; protein and metabolite transport; and key metabolic pathways such as Fe-S cluster biosynthesis, branched chain amino acid degradation, glycine cleavage, ubiquinone biosynthesis and fatty acid oxidation. Mitoproteins participating in protein folding, processing and degradation, as well as regulation and metabolism of reactive oxygen species are also prominent, and the wide array falling into the six Enzyme Commission (EC) classes testifies to a robust organellar metabolism.

Although the functionally most important components of various mitochondrial complexes were identified in our study, we failed to retrieve homologs of a number of subunits that are present in other discobids, notably *Andalucia.* Examples are various ETC accessory proteins, which tend to be less well conserved at the sequence level, making a definitive identification challenging. While it is possible that some of these ‘missing’ mitoproteins are among the large number of uncharacterized members listed in category J (Supplementary Table S2), it is notable that they also appear to be absent from available kinetoplastid and euglenid proteome data. On the other hand, the *D. papillatum* mitoproteome contains homologs of proteins initially identified as additional novel components in trypanosomatids, e.g., of the ETC complexes (Table 1). Recruitment of new proteins to mitochondrial complexes, which applies also to other features such as mitoribosome assembly and structure and the atypical ATOM complex, is evidently a euglenozoan trait, as homologs of some of these novel proteins have also been reported in the *E. gracilis* mitoproteome [22]. These observations imply that re-tailoring of mitochondrial complexes through addition of new subunits must have been underway already in the last euglenozoan common ancestor. Lineage-specific re-tailoring has evidently continued *via* loss of some of these novel proteins and gain of others within the various euglenozoan clades, as evidenced by proteomic analyses that have identified novel proteins specific to *Diplonema* in CI and the mitoribosome of this protist [14, 27].

In addition to mitochondrial re-tailoring through new acquisitions, *D. papillatum* also encodes multiple paralogs of various components, notably ATP synthase subunits, as well as proteins implicated in mitochondrial ETC assembly, membrane sculpting, and protein trafficking and turnover. Those that were detected *via* proteomics display different abundances, which suggests the possibility that alternative sets of paralogs are differentially expressed depending on nutritional, environmental or other conditions, hence specializing in their roles.

A primary aim of our mitoproteome analysis was to identify proteins that might be implicated in the extensive RNA processing that occurs in *Diplonema* mitochondria, which involves many transcript ligation and editing steps. We were successful in identifying several prime candidates for carrying out the various enzymatic steps in the pathway: an RtcB-like RNA ligase (RTCB1) in ligation of transcript modules; two TUTases in U-appendage RNA editing; and a DYW member of the PPR protein family in deamination of C and A residues. We posit that the unusually large family of PPR proteins (~ 100) may serve as site-specificity factors for deamination editing, as has been documented in land plant mitochondria. Several other multi-protein families of RNA binding proteins could conceivably facilitate transcript ligation; these families include DnaJ domain-containing proteins (30 members); DExD/H-box RNA helicases (11 members); and at least seven proteins exhibiting a CSD domain.

The availability of the first comprehensive mitoproteome data for a diplonemid, in conjunction with published mitoproteome data for other members of Discoba, allows us to draw certain inferences about mitochondrial evolution within this clade. Considering the *A. godoyi* mitoproteome, with its strikingly bacteria-like features, as reflective of the mitoproteome of the last discobid common ancestor, some profound changes have evidently occurred in the transition from this ancestor to the last euglenozoan common ancestor. Major changes include transition from a bacteria-like rdxPolIA DNAP to a different PolIA; replacement of the ancestral mtDNA-encoded, multi-subunit, bacteria-like RNAP with a nucleus-encoded, single-subunit, virus-like RNAP; and loss of a number of bacteria-like pathways, notably the FtsZ-Min machinery involved in cell division in bacteria. Additional functions present in *Andalucia* but absent in euglenozoans include a type 2 protein secretion system (T2SS), a twin-arginine translocation (TAT) pathway, a Ccm cytochrome *c* biogenesis system, and a three-component aerobic-type rubrerythrin system. Other bacteria-like features identified in the *A. godoyi* mitoproteome but not in any available euglenozoan mitoproteome include a bacterial-type GreA/GreB transcription elongation/transcript cleavage factor, which complements the bacterial-type mitochondrial RNAP; RnpA, the protein component of bacterial RNase P; and a bacterial-type RecA. The one bacterial feature retained in *D. papillatum* is an HslVU protease, also preserved in other euglenozoans.

Moreover, re-tailoring of the ETC complexes and the mitoribosome by loss of some conventional subunits and addition of numerous novel ones must have begun before the emergence of the last euglenozoan common ancestor, given that homologs of certain novel subunits are identifiable in all three euglenozoan clades. Gene fragmentation concomitant with transfer from the mitochondrial to the nuclear genome is another notable euglenozoan feature. A split Sdh2 gene with separate nucleus-encoded N-terminal and C-terminal ‘half-genes’ has been characterized in *E. gracilis* [32], *T. brucei* [33] and *D. papillatum* (this report), while the mtDNA-encoded Sdh2 gene is intact in *A. godoyi* [5]. The same situation applies to the Nad11 gene, although in this case only the N-terminal portion of the corresponding protein appears to have been retained as a nucleus-encoded half-gene in all three euglenozoan clades. The fate of the C-terminal moiety has so far been elucidated only in *Euglena*, where it appears to have been substituted by recruitment of a new protein with Nad11-like structure [30], which nevertheless lacks counterparts in both kinetoplastids and diplonemids.

Evolution of the euglenozoan mitoproteome has evidently taken different paths within the individual lineages, after their divergence from the last euglenozoan common ancestor and each other. While euglenids retain a PolIA DNAP, this enzyme was replaced by a virus-derived PolIBCD+ DNAP in the common diplonemid-kinetoplastid ancestor. As well, because almost all of the mitochondrial-type aminoacyl-tRNA synthases present in euglenids are absent in both diplonemids and kinetoplastids, the loss presumably preceded their separation from a common ancestor. Re-tailoring of mitochondrial complexes has evidently continued within the individual clades, as evidenced by clade-specific additions leading, e.g., to distinct compositions of the diplonemid and kinetoplastid ETC complexes and mitoribosomes.

Finally, what we might term the ‘dark’ mitoproteome—that portion whose function is unknown—is an intriguing feature of every mitoproteome that has been characterized to date. In *D. papillatum*, over one-third of candidate mitoproteins fall into this category, with 20% of the overall mitoproteome comprising diplonemid-specific proteins having homologs only in other diplonemid species and a further 10% seemingly exclusive to *D. papillatum*. The existence of corresponding transcripts and in some cases mass spectrometry data (Fig. 1) coupled with the presence of homologs in multiple diplonemid species argues strongly that these inferred unknown function proteins are indeed authentic.

Direct experimentation will be necessary to reveal the sub-mitochondrial localization and role(s) of the hundreds of *Diplonema*-specific mitoproteins identified here. Some, perhaps many, may represent proteins that have been recruited specifically to diplonemid mitochondrial complexes, as has been demonstrated by proteomic analysis in the case of ETC CI [27] and the mitoribosome [14]. The RNA processing complexes that mediate the extensive RNA ligation and editing events in *Diplonema* mitochondria, as detailed above, are likely candidates for the location and function of some of the additional unknown proteins listed here.

## Materials and methods

### Datasets of inferred diplonemid proteomes

The collection of diplonemid proteins (≥ 100 residues) used in this study comprises more than 1.54 million proteins. These include 37,343 genome and transcriptome-inferred proteins from *D. papillatum* [24] along with transcriptome-derived proteome sequences from 11 other diplonemids. The species and protein counts (in parentheses) are as follows: *Artemidia motanka* (117,812), *Diplonema aggregatum* (131,319), *Diplonema ambulator* (134,348), *Diplonema japonicum* (101,797), *Flectonema neradi* (143,870), *Hemistasia phaeocysticola* (140,521), *Lacrimia lanifica* (139,225), *Namystynia karyoxenos* (146,560), *Rhynchopus euleeides* (209,261), *Rhynchopus humris* (80,007), and *Sulcionema specki* (164,075) [12]. For the analyses reported here, the *D. papillatum* protein set initially published in NCBI’s bioprojects (https://identifiers.org.bioproject:PRJNA883718) was further enhanced by removing spurious sequences that originated from the conceptual translation of assembled transcripts in multiple reading frames (available at FigShare at 10.6084/m9.figshare.c.8041126.v1). Furthermore, ~ 6,000 gene models that included repetitive elements and lacked evidence for transcription were removed from the *D. papillatum* proteome used in this study.

### Prediction of mitochondrial protein localization

To predict mitochondrial protein localization, we tested six tools that could be run locally, namely DeepLoc 2.0 [159], MULocDeep v1 [160], WoLF PSORT v0.2 [161], TargetP2 v2.0 [162], MitoFates v1.2 [163] and MitoProtII v1.101 [164]. We tested these tools on manually curated sets of true positives (469 sequences) and true negatives (482), representing *D. papillatum* nucleus-encoded proteins known to be mitochondrial or non-mitochondrial, respectively, mostly based on previous research (e.g [24, 26–28]).,. The latter three tools, which predict mitochondrial localization specifically, only marginally improved the true rates compared to the former three tools. Consequently, we opted to use DeepLoc 2.0 (here referred to as DeepLoc2), MULocDeep, and WoLF PSORT because of their good performance on the reference data. All three tools predict subcellular localization, not only mitochondrial localization, which might contribute to their higher accuracy. Based on the true and false positive and negative rates, we devised a formula to combine the predictions of the three selected tools and calculated a weighted average (WA) score for each protein: *[(MULocDeep ‘mitochondrion’ score × 2.5) + (normalized WoLF PSORT ‘mitochondrion’ score × 1.5) + (DeepLoc2 ‘mitochondrion’ score × 6)] ÷ 10*. (The score generated by WoLF PSORT was normalized because in contrast to the other tools, the values it returns fell outside the 0–1 range.) DeepLoc2 performed best on our reference data, and all proteins assigned to the ‘Mitochondrion’ by this tool (WA score range 0.25–1) were considered to be mitochondrial. More precisely, we classified as “likely mitochondrial” all sequences falling in the WA score range of 0.25–0.49 (corresponding to a 8:1 likelihood of being mitochondrial rather than non-mitochondrial based on true positive and negative identification rates), as “very likely mitochondrial” at WA scores 0.5–0.74 (27:1 likelihood of being mitochondrial), and as “almost certainly mitochondrial” those at WA scores 0.75–1. Scores of all proteins that DeepLoc2 classified as non-mitochondrial, but for which MULocDeep (the tool performing as the second best on the test data) inferred a mitochondrial localization, were downgraded by a factor of 0.25, so that their penalized WA scores fell within the range of 0.01–0.24. We considered these sequences to have a “low probability of mitochondrial localization” with the likelihood of ~ 1:3 to be mitochondrial). Finally, WA scores were defaulted to 0 for all proteins classified as non-mitochondrial by both DeepLoc2 and MULocDeep (< 1:41 likelihood of being mitochondrial).

### Analyses of mass spectrometry data

The primary resource for classifying a protein as mitochondrial vs. non-mitochondrial was the PRIDE database dataset PXD035104, which allowed us to determine the protein level in whole cell lysates, cytosol, and enriched mitochondria of *D. papillatum* [14, 26, 27]. Prior to data analysis, the Thermo RAW format was converted to mzML using ThermoRawFileParser v1.3.2 [165]. Peptide searches in the raw MS/MS datasets were performed using MSFragger v3.5 [166], followed by filtering, scoring, and quantification by Philosopher v4.4.0 [167] and IonQuant v1.8.0 [168]. The mitochondrial enrichment was calculated as follows: (1) for each protein, the sum of spectral counts (or ion intensities) in the mitochondrial and cytosolic fraction was divided by the sum of spectral counts (or ion intensities) in whole cells, except when no peptide was detected in the whole-cell lysate, in which case the divisor was set to the minimal non-zero value in the dataset, i.e., 1 for spectral counts and 35,000 for ion intensities, (2) if the ‘mitochondrion : whole cell’ ratio was larger than the ‘cytosol : whole cell’ ratio and simultaneously > 1.5, the protein was considered as ‘detected in mitochondria’. Proteins were quantified by calculating iBAQ values as described previously [14, 26, 27]. In addition, all proteins detected via proteomics in the respirasome [27] and in the mitoribosome [14] of *D. papillatum* were classified as ‘detected in mitochondria’ by association to these macromolecular complexes.

### Protein phylogenies

To capture the diversity of the RtcB protein family, proteins with sequence similarity to the three *D. papillatum* RTCB proteins were collected by BLAST [169] searches in the GenBank nr repository on March 22, 2024, and by diamond [170] searches in a local protist database combining the EukProt-v3 collection [171], a Discoba-centric sequence collection [172], and the 12 available transcriptome-inferred diplonemid proteomes [12]. GenBank sequences were first aligned with MAFFT v7.490 at default parameters [173] to identify intein segments, which were removed, and then clustered at 80% using CD-HIT [174]. From the RTCB matches in the local protist database, we first removed sequences shorter than 305 amino acids (80% of the shortest RtcB family protein known from literature), then aligned the remaining sequences with MAFFT v7.490 [173] to identify clearly truncated sequences, which were removed as well. To identify likely bacterial contaminants present in the EukProt-v3 collection, the alignment was analyzed by FastTree v2.1.11 at default parameters [175], and all candidate contaminants were examined by BLAST searches against GenBank; if the identity to a bacterial sequence was > 98%, the candidate contaminant was removed. All protist hits except from diplonemid sequences were then clustered at 80% using CD-HIT. The collection was manually supplemented by 10 distinct RtcB proteins whose structures or biochemical activities had been analyzed in more detail, namely those from *Pyrococcus horikoshii* (one sequence), *Thermus thermophilus* (one sequence), *Myxococcus xanthus* (six sequences), and *Escherichia coli* (two sequences). In total, our RtcB family collection contained 1,183 sequences (662 from bacteria, 91 from archaea, 25 from viruses, and the rest from eukaryotes, out of which protist sequences represented ~ 95%). Proteins were aligned with Clustal Omega v1.2.3 at default parameters [176] and positions with > 90% gaps were removed. The phylogenetic tree was constructed by IQ-Tree v2.3.4 [177] using the model NQ.pfam + F + R15, which was selected by the program as the most suitable for the dataset, and performing 1,000 replicates for the SH approximate likelihood ratio test and 2,000 replicates for ultrafast bootstrap. The full tree (in the Newick and PDF formats), as well as the collection of sequences are available at FigShare at 10.6084/m9.figshare.c.8041126.v1.

### Search for structural homologs

We used Foldseek [178] to search proteins in the *D. papillatum* proteome that resemble in their three-dimensional structure RET1 [137] and RET2 [138] from *Trypanosoma brucei*, *T. brucei*-specific components of respiratory chain complexes (based on the UniProt-AlphaFold release UP000008524_185431_TRYB2_v4), and respirasome components of *Euglena gracilis* [30]. Conversely, structures of *D. papillatum*-specific Complex I proteins [27] predicted with AlphaFold2 [179] and OmegaFold v2 (https://github.com/HeliXonProtein/OmegaFold) were used as reciprocal queries for searches in the *T. brucei* UniProt-AlphaFold release v4. The dataset of predicted structures of the entire *D. papillatum* proteome was released recently [180].

### Search for Pfam domains

To identify *D. papillatum* proteins that potentially play a role in mitochondrial RNA editing, we screened the inferred proteome for the occurrence of 10 Pfam domains known from other systems to be involved in similar processes. As a negative control, we also searched five domains specific to poly(A) polymerase (Supplementary Table S3). The corresponding profile HMMs were retrieved from PFAM-A using hmmfetch (Easel library 0.48) and employed in searching the proteome with hmmsearch [181] using the options -max and --E 0.05.

### Expert-validated reference datasets of PPR proteins and motifs from *D. papillatum*

To assess the performance of hidden Markov model (HMM) searches described below, we used expert-validated reference datasets from *D. papillatum* (available at FigShare at 10.6084/m9.figshare.c.8041126.v1). These datasets were established by employing various resources and methods, such as the TPRpred webserver [182], three-dimensional structure analysis with Alphafold2 [183, 184], search with profile HMMs built by phmmer [181] from function-known proteins of model organisms, and visual inspection of multiple protein alignments generated with Muscle [185]. The positive PPR-protein reference comprised 106 expert-validated *D. papillatum* sequences. The positive PPR-motif reference, with 79 sequences, was obtained by selecting those motifs contained in the protein reference that were retrieved in HMMER searches with an independent E-value (i-E-value) match of < 10 to plant P-type PPR-motifs (see next section). The negative motif reference contained 1,457 expert-validated non-PPR repeat motifs from 247 *D. papillatum* proteins. This latter category included TetratricoPeptide Repeats (TPRs) and Ankyrin repeats, which, due to their similar length and 3-dimensional structure as PPR motifs, are susceptible to misidentification through automated procedures.

### Search with profile HMMs

HMM searches were performed for protein families such as the DnaJ and DExD/H-box families as well as for PPR proteins. The former category was initially identified by blast searches and used for building profile HMMs. With these profiles, an HMM search in the inferred *D. papillatum* mitoproteome was performed with hmmsearch v3.3.0 [181] using --notextw --domE 100 --domtblout, and otherwise default parameters. To identify PPR proteins, we searched across the combined inferred proteomes from diplonemids, first by using profile HMMs of the plant PPR-motif subclasses P, P1, P2, L1, L2, S1, S2, SS, E1, and E2 (i.e., excluding DYW, https://ppr.plantenergy.uwa.edu.au/). For the second iteration, we searched with the diplonemid P-type PPR profile that was built in house (see Supplemental Methods Section ‘Construction of a diplonemid-specific profile HMM’).

### Classification performance and threshold definition for selecting reliable PPR motifs

We tested the performance of the HMM search with plant profiles by assessing how well PPR and non-PPR motifs were distinguished using the above-described curated motif reference datasets. As a scoring metric, we used the i-E-value reported by hmmsearch for each particular domain. The accuracy of predictions was assessed by calculating precision (P) and recall (R), defined as.

P = TP/(TP + FP).

R = TP/(TP + FN).

with TP being true positives, FP false positives, and FN false negatives. The F1 score, calculated as the harmonic means of precision and recall, is defined as.

F1 = TP/(TP + 0.5(FP + FN)).

F1 values were plotted against various E-value thresholds (Supplementary Figure S4) to choose the largest E-value at which the HMM search retrieved all positive reference PPR motifs but none of the negative reference motifs. This E-value was then used as a threshold to identify motifs with high confidence among all those retrieved by the HMM search. The applied motif E-value threshold was 8.6 for the first iteration (performed with the plant profile HMMs) and 11 for the second (performed with the diplonemid-specific profile HMM).

### Identification of PPR-motif candidates based on location and structure analysis

To identify PPR motifs in *D. papillatum* proteins that are part of a tandem motif repeat array, but may have been missed by HMM searches, the HMM output was screened for 30–40 residue-long gaps between identified motifs. For validating PPR-motif candidates found in gaps, the corresponding sequences plus 10 residues up and downstream were extracted, and their potential secondary (2D) structure was analyzed with a script that makes use of the NetsurfP-3.0 webserver at https://services.healthtech.dtu.dk/services/NetSurfP-3.0/ [186]. Structural signatures specific to PPR motifs were derived from the Alphafold2 3-state 2D structure prediction [183, 184] of 18 reference proteins listed in Supplementary Table S4. These signatures served as the basis to formulate the following structural criteria defining PPR motifs: both helices must contain a minimum of seven helix-forming residues; non-helical amino acids are permitted within helical regions; and three consecutive ‘turn’ residues must be present at positions 11 to 13 within the motif. Examples of secondary structures meeting these criteria are provided in Supplementary Table S5.

### Construction of a diplonemid-specific profile HMM

To construct a specific profile HMM for diplonemid P-type PPR motifs, two sets of motifs were combined. The first set comprised ~ 1,500 high-confidence motifs from nearly 1,000 proteins identified through the HMM search with plant models in diplonemid proteomes (for the i-E-value threshold, see the Section ‘Classification performance’). The second set consisted of validated candidate motifs found within gaps between assigned PPR motifs of *D. papillatum* proteins, motifs that met the structure criteria outlined above. These two sets of PPR motif sequences were combined and aligned with Muscle v3.8.1551 [185], and the resulting multiple sequence alignment served for building the diplonemid-specific P-type profile HMM using hmmbuild [187].

### Custom scripts

Scripts developed in the context of this study have been deposited on GitHub at the URL https://github.com/FelixLeSieur/PPR-project.

## Supplementary Information

Supplementary Material 1.

Supplementary Material 2.

Supplementary Material 3.

## Data Availability

Supplementary Material is available with this article. Datasets of inferred diplonemid proteomes, the updated *D. papillatum* proteome from which spurious and repetitive element-derived sequences have been removed, the complete phylogenetic tree of RTCB proteins, all RTCB protein sequences, the reference datasets of PPR proteins, as well as copies of the figures, supplementary files, and supplementary tables can be accessed through the online data repository FigShare at 10.6084/m9.figshare.c.8041126.v1.
