# Supplementary material for: The mitochondrial proteome of diplonemids: from conventional pathways to eccentric RNA editing and transcript processing
Source: BMC Genomics. 2025 Dec 11;26:1099. doi: 10.1186/s12864-025-12233-1 (PMC12699882; doi:10.1186/s12864-025-12233-1)
Supplement: Supplementary file 3 — Supplementary Material 3. [file 12864_2025_12233_MOESM3_ESM.pdf]

The Mitochondrial Proteome of Diplonemids:
from Conventional Pathways to Eccentric RNA Editing and Transcript Processing

Michael W. Gray[1], Matus Valach[2], Matt Sarrasin[2], Felix-Antoine Le Sieur[2], Julius Lukeš[3,4], Gertraud Burger[2]

[1]Department of Biochemistry and Molecular Biology and Institute for Comparative Genomics, Dalhousie University, Halifax, NS, Canada

[2]Département de Biochimie and Robert-Cedergren Center for Bioinformatics and Genomics, Université de Montréal, Montréal, QC, Canada

[3]Institute of Parasitology, Biology Centre, Czech Academy of Sciences, České Budějovice
(Budweis), Czechia

[4]Faculty of Sciences, University of South Bohemia, České Budějovice (Budweis), Czechia

Supplementary File S1

Contents

Results

Identification of *Diplonema papillatum* PPR proteins

Supplementary Figures

Supplementary Figure S1, Diplonemid RTCB homologs have all important catalytic residues.

Supplementary Figure S2. Candidate diplonemid mitochondrial TUTases have all catalytic residues.

Supplementary Figure S3. The DYW_deaminase domain of diplonemid DIPPA_21411 homologs carries the critical catalytic residues.

Supplementary Figure S4. Determination of the E-value cutoff for selecting high-confidence PPR motifs among the ones retrieved by default profile-HMM searches.

Supplementary Tables

Supplementary Table S1. *D. papillatum* proteins predicted to be targeted to and localized in mitochondria (i.e., mitoproteins). [*Separate Excel file*]

Supplementary Table S2. Annotated categories of predicted *D. papillatum* mitoproteins. [*Separate Excel file*]

Supplementary Table S3. Pfam protein domains used to search the *Diplonema* proteome.

Supplementary Table S4. Proteins used to derive structural signatures of PPR motifs.

Supplementary Table S5. Secondary structure criteria for validating PPR motifs.

References

Results

Identification of *Diplonema papillatum* PPR proteins

Identifying pentatricopeptide repeat (PPR) proteins is challenging due to their complex structure, comprising multiple tandem repeat motifs of diverse sequence, making traditional methods like BLAST, which rely on pairwise sequence alignment, ineffective. Therefore, we employed profile HMM searches using models built from PPR *motifs* rather than for entire proteins. With these profiles, we searched in the collection of diplonemid proteomes consisting of the quasi-complete genome-inferred proteome of *D. papillatum* [1] and the partial transcriptome-derived proteomes of 11 other diplonemids [2].

An initial search with plant PPR HMM motifs including 10 subclasses (P, P1, P2, L, etc.; see Supplemental Methods) retrieved a total of ~12,000 motifs in around 2,000 diplonemid proteins including 134 high-confidence motifs in 87 distinct *D. papillatum* proteins (see Supplemental Methods for the E-value threshold applied). The large majority (80%) and the strongest hits were obtained with the P, P1, and P2 motifs. In many instances, matches with P, P1 and P2 overlapped, so that the distinction established for plant P-type motifs seems not to apply to diplonemids. A small number of L- and S-type motifs was detected as well, but these were not arranged in tandemly repeated P-L-S triplets as known from plants [3].

A detailed examination of the HMM-search results for *D. papillatum* revealed 30-40-residue long 'gaps' between the assigned PPR motifs. Secondary structure inspection revealed that 15 of these gaps shared characteristics with validated PPR motifs available in SwissProt. Given these similarities, we consider the detected 'gaps' as novel, divergent types of PPR motifs.

To uncover additional PPR proteins within the *D. papillatum* proteome, we developed a single, diplonemid-wide PPR-motif profile HMM and performed a second search iteration. The model was built with high-confidence P-type motifs from diplonemids along with the newly detected structure-validated 'gap' motifs from *D. papillatum*. The search with this profile HMM identified in the *D. papillatum* proteome 147 additional PPR motifs in a total of 102 proteins. Combining the search results of the first and second iterations, we determined 281 distinct high-confidence PPR-motifs in 121 distinct *D. papillatum* proteins. As anticipated, the majority (108) of these proteins are of predicted mitochondrial location (Supplementary File S2, category C and D). For details, see 'Prediction of mitochondrial protein localization' in METHODS, below.

Surprisingly, in approximately 40% of the retrieved proteins, only a single PPR motif was located. We expect that the seemingly single-motif proteins include deviant PPR motifs that remained undiscovered due the stringent E-value threshold and the limited number of search iterations. Before further extending the computational search, it will be essential to experimentally confirm the RNA-binding potential and protein-RNA interactions of select PPR motifs.

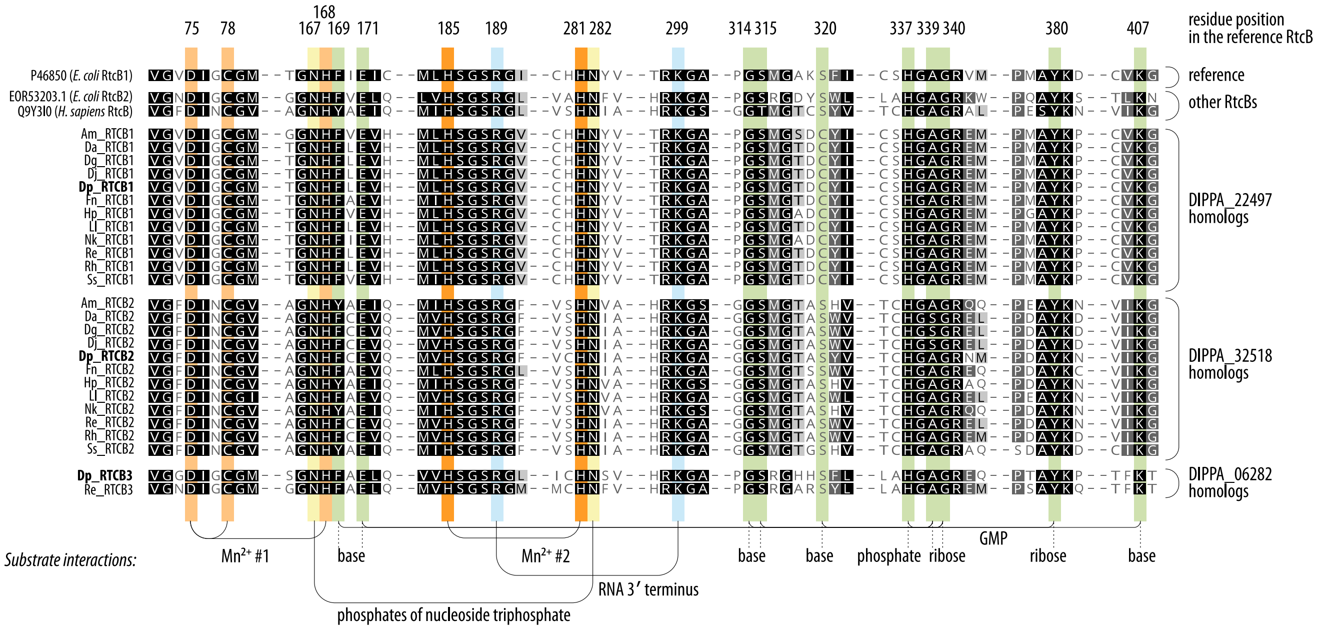

Supplementary Figure S1. Diplonemid RTCB homologs have all important catalytic residues. Multiple sequence alignment of diplonemid RTCB homologs with experimentally validated proteins of the RtcB-family. Reference: RtcB, *Escherichia coli* RtcB1 (P46850). Dark and light grey shading indicates higher and lower amino acid conservation, respectively. The region shown corresponds to amino acids 72–408 of the reference, which contains the catalytically critical residues involved in the binding of two manganese ions (Mn^{2+}; light and dark orange), the RNA substrate terminus (blue), phosphates of the substrate nucleotide triphosphate (yellow), and the nucleoside moiety of guanosine monophosphate (GMP; green). Diplonemid species abbreviations: Am, *Artemidia motanka*; Da, *Diplonema ambulator*; Dg, *D. aggregatum*; Dj, *D. japonicum*; Fn, *Flectonema neradi*; Hp, *Hemistasia phaeocysticola*; Ll, *Lacrimia lanifica*; Nk, *Namystynia karyoxenos*; Re, *Rhynchopus euleeides*; Rh, *R. humris*; Ss, *Sulcionema specki*.

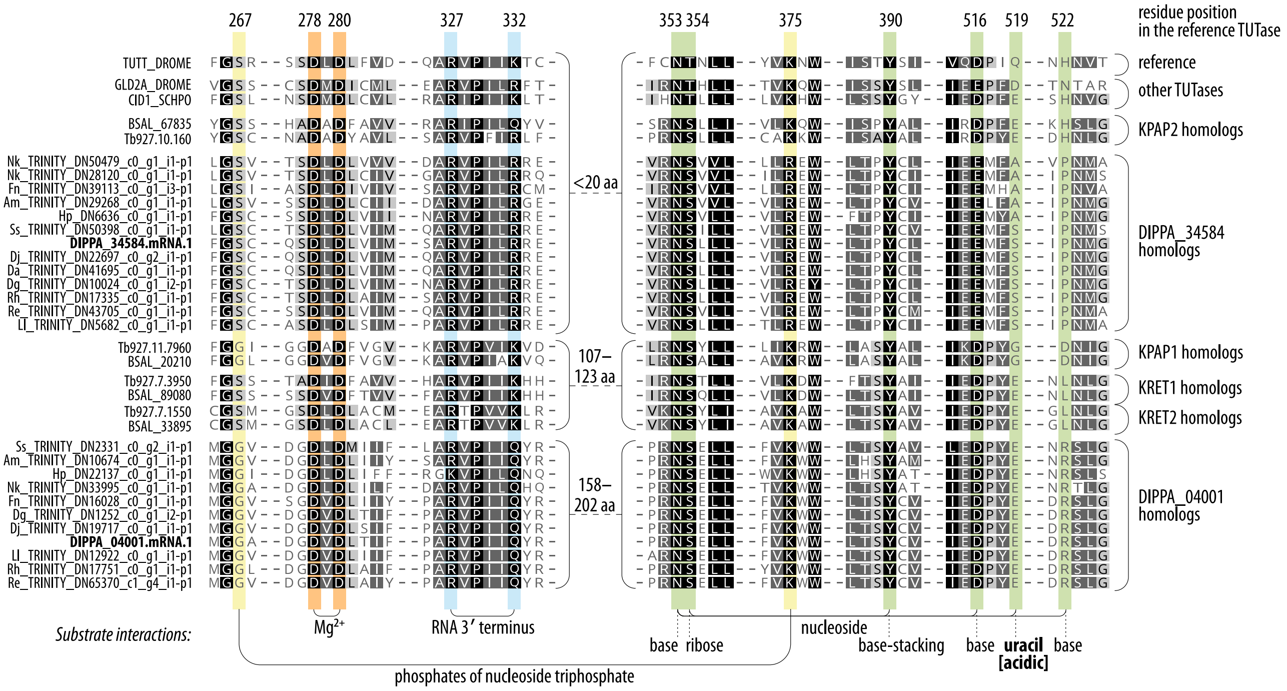

Supplementary Figure S2. Candidate diplonemid mitochondrial TUTases have all catalytic residues. Multiple sequence alignment of diplonemid mitochondrial proteins belonging to the terminal nucleotidyl transferase family with experimentally confirmed TUTases and poly-A polymerases. References: *Drosophila melanogaster* TUTase Tailor (TUTT_DROME); KPAP1, KPAP2, kinetoplastid mitochondrial poly-A polymerases; KRET1, KRET2, kinetoplastid mitochondrial RNA editing TUTases. The region shown corresponds to amino acids 265–525 of TUTT_DROME. Dark and light grey shading indicates higher and lower conservation, respectively. The protein segments shown contain the catalytically critical residues involved in the binding of magnesium ions (Mg^{2+}; orange), the RNA substrate terminus (blue), the terminal phosphate of the substrate nucleotide triphosphate (i.e., UTP in TUTases or ATP in PAPs; yellow), and the nucleoside moiety of the substrate nucleotide triphosphate (green). Note that TUTases have a distinctive acidic residue at position 519, which binds the leaving UMP. In PAPs, this residue is usually non-acidic. Diplonemid homologs of DIPPA_04001 carry long insertions in the region between the residues 334 and 351 of the reference, otherwise a typical feature of KRET family proteins, where it is postulated to be involved in mediating protein-protein interactions in the RNA editing complex of kinetoplastids. For diplonemid species abbreviations, see legend to Supplementary Figure S1.

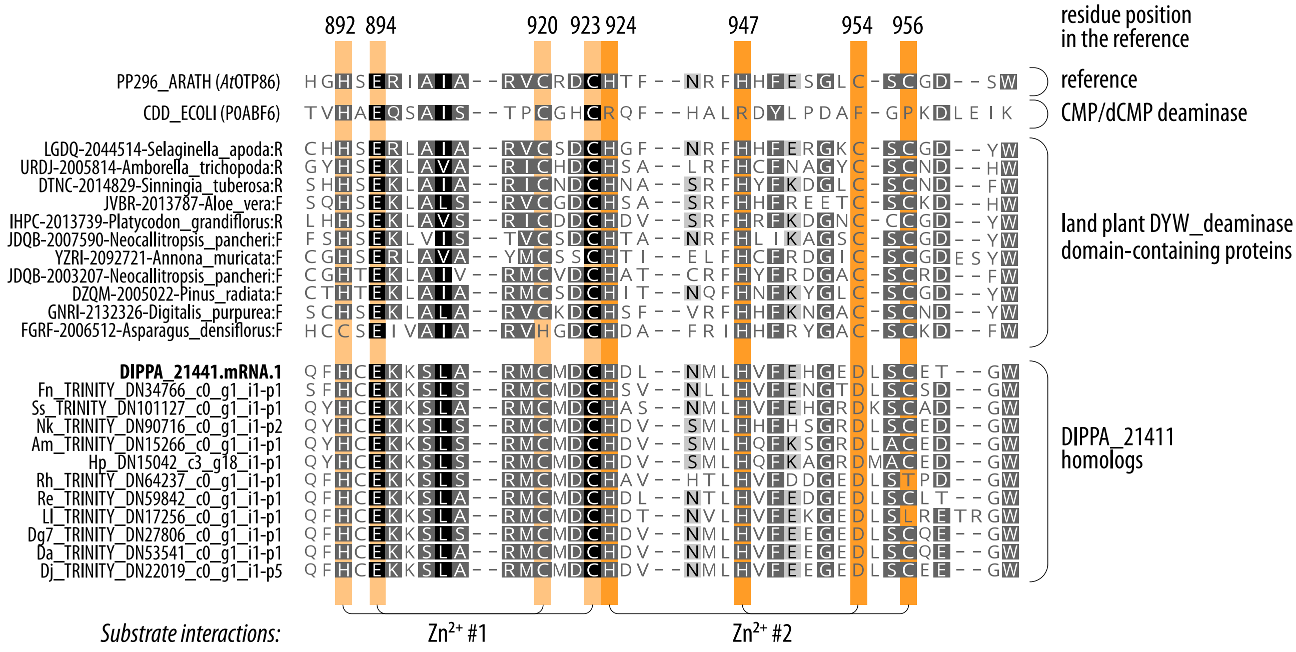

Supplementary Figure S3. The DYW_deaminase domain of diplonemid DIPPA_21411 homologs carries the critical catalytic residues. Multiple sequence alignment of diplonemid proteins of the DYW_deaminase family with experimentally validated deaminases. Dark and light grey shading indicates higher and lower amino acid conservation, respectively. Reference DYW_deaminase-family protein – *Arabidopsis thaliana* OTP86 (PP296_ARATH). The region shown corresponds to amino acids 890–960 of the reference and includes the residues critical for the binding of two zinc ions (Zn^{2+}; light and dark orange highlight bars). Two deviations are noteworthy. First, at the reference position 954, the Cys typical for the structural Zn ion binding site (Zn^{2+} #2) of plant DYW deaminases has been substituted in diplonemid counterparts by an Asp residue that is potentially catalytic (e.g., [4, 5]). Second, the widely conserved Cys in position 956 is replaced by Thr and Leu in *R. humris* and *L. lanifica*, respectively. Note that the deaminase of free CMP/dCMP from *E. coli* (CDD_ECOLI, P0ABF6) completely lacks the second Zn ion-binding site. Note also that the eponymous 'DYW' motif (958–960 in the reference) is not involved in the catalysis nor conserved even across plants. For diplonemid species abbreviations, see legend to Supplementary Figure S1.

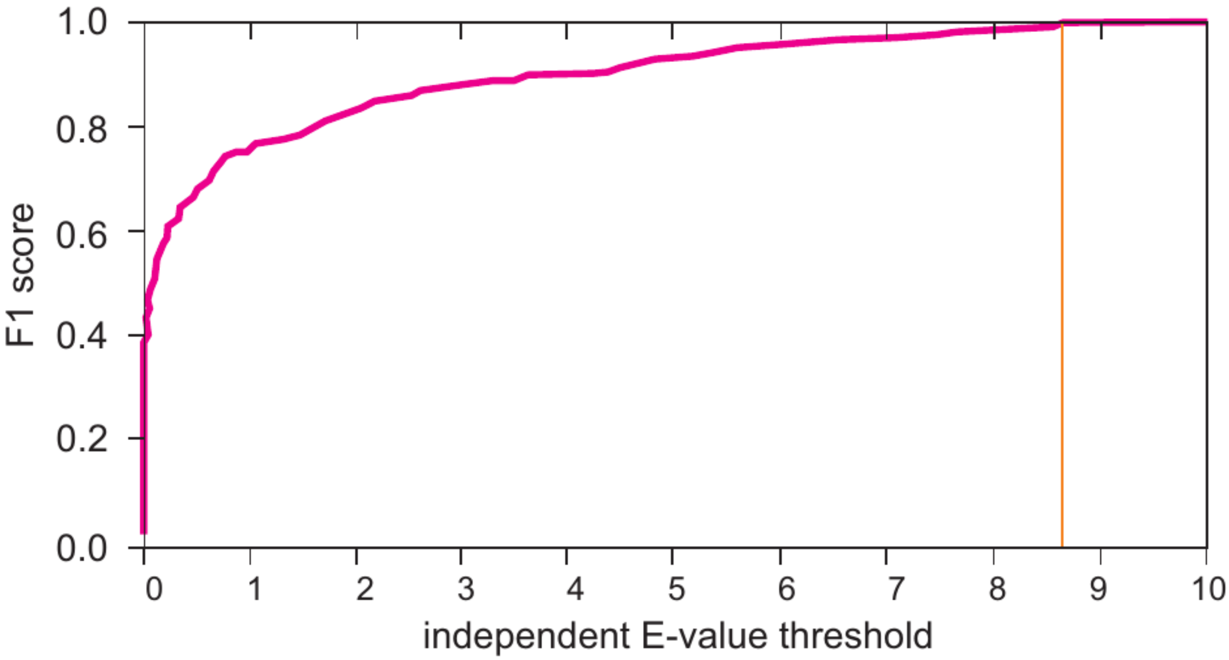

Supplementary Figure S4. Determination of the E-value cutoff for selecting high-confidence PPR motifs among the ones retrieved by default profile-HMM searches. From the output of the search with a PPR-motif profile HMM against the combined diplonemids proteomes, the reported independent E-values (i-E-values) of expert-validated positive and negative reference motifs were collected. Then, the corresponding F1 values were calculated for each E-value threshold from 0 to 10 and plotted against the i-E-value (see Methods). In the search with plant PPR profiles shown here, the i-E-value at which all positive and none of the negative reference motifs were retrieved, was 0.86 (see orange vertical line). This value was chosen as an upper threshold for high-confidence PPR motifs.

Supplementary Table S1. ***D. papillatum* proteins predicted to be targeted to and localized in mitochondria (i.e., mitoproteins).** A separate Excel file lists weighted average (WA) scores and associated data based on the procedure outlined in the Material and Methods section. Only entries having a WA score >0.5 were considered candidate mitoproteins in this study.

Supplementary Table S2. Annotated categories of predicted *D. papillatum* mitoproteins. A separate Excel file lists candidate mitochondrial proteins from *D. papillatum* and relevant information about them, including WA scores and amino acid sequences. The Key tab defines various aspects of the presentation (color coding, etc.). Functional categories as defined in Fig. 1 are organized in subsequent tabs (A) through (J), with relevant statistical data compiled in the final (Statistics) tab.

Supplementary Table S3. Pfam protein domains used to search the *Diplonema* proteome.

Pfam Accession[a]	Pfam ID	Description
PF19088	TUTase	TUTase nucleotidyltransferase domain
PF16631	TUTF7_u4	Unstructured region 4 on terminal uridylyltransferase 7
PF00483	NTP_transferase	Nucleotidyl transferase
PF18528	Ret2_MD	RNA editing 3' terminal uridylyl transferase 2 middle domain
PF03828	PAP_assoc	Cid1 family poly A polymerase
PF18774	APOBEC4_like	APOBEC4-like -AID/APOBEC-deaminase
PF14432	DYW_deaminase	DYW family of nucleic acid deaminases
PF02295	z-alpha	Adenosine deaminase z-alpha domain
PF08210	APOBEC_N	APOBEC-like N-terminal domain
PF18769	APOBEC1	APOBEC1
PF02137	A_deamin	Adenosine-deaminase (editase) domain
PF01743	PolyA_pol	Poly A polymerase head domain
PF12626	PolyA_pol_arg_C	Polymerase A arginine-rich C-terminus
PF12627	PolyA_pol_RNAbd	Probable RNA and SrmB- binding site of polymerase A
PF04926	PAP_RNA-bind	Poly(A) polymerase predicted RNA binding domain
PF04928	PAP_central domain	Poly(A) polymerase central

[a] Pfam at InterPro 98.0

Supplementary Table S4. Proteins used to derive structural signatures of PPR motifs.

UniProtID[a]	Protein Name	Description
Q9FME4	PP438_ARATH	Small ribosomal subunit protein mL104 (rPPR9)
Q0WMY5	PP365_ARATH	Pentatricopeptide repeat-containing protein At5g04810, chloroplastic
B8Y6I0	PPR10_MAIZE	Pentatricopeptide repeat-containing protein 10, chloroplastic
A7LN87	PPR5_MAIZE	Pentatricopeptide repeat-containing protein PPR5, chloroplastic
O42955	CCM1_SCHPO	Pentatricopeptide repeat-containing protein 3, mitochondrial
Q9LSQ2	PP239_ARATH	Putative pentatricopeptide repeat-containing protein At3g16890, mitochondrial
O42955	CCM1_SCHPO	Pentatricopeptide repeat-containing protein 3, mitochondrial
Q96EY7	PTCD3_HUMAN	Small ribosomal subunit protein mS39
Q8L844	PP413_ARATH	Pentatricopeptide repeat-containing protein At5g42310, chloroplastic
Q66GI4	PRRP1_ARATH	Proteinaceous RNase P 1, chloroplastic/mitochondrial
Q0WNP3	PP319_ARATH	Pentatricopeptide repeat-containing protein At4g18520, chloroplastic
P0C7R1	PPR74_ARATH	Pentatricopeptide repeat-containing protein DWY1, chloroplastic
Q9SY69	PPR29_ARATH	Pentatricopeptide repeat-containing protein At1g10270
Q9FWA6	PP207_ARATH	Pentatricopeptide repeat-containing protein At3g02330, mitochondrial
Q9M9E2	PPR45_ARATH	Pentatricopeptide repeat-containing protein At1g15510, chloroplastic
Q0WSH6	PP312_ARATH	Pentatricopeptide repeat-containing protein At4g14850
Q8GWE0	PP314_ARATH	Pentatricopeptide repeat-containing protein At4g16390, chloroplastic
O22137	PP202_ARATH	Pentatricopeptide repeat-containing protein At2g45350, chloroplastic

[a] UniProtKB release 2024_01

Supplementary Table S5. Secondary structure criteria for validating PPR motifs.

ProteinID	Motif start – end	Structure	Evaluation[a]
DIPPA_05641.mRNA.1	19 –54	+HHHHHHHHHHHH++++HHHHHHHHHHHH+++++++	Valid
DIPPA_12895.mRNA.1	297 –331	HHHHHHHHHHHH+++HHHHHHHHHHHHH+++++H	Valid
DIPPA_07167.mRNA.1	212 –246	HHHHHHHHHHHHH++HHHHHHHHHHHH++++++H	Invalid
DIPPA_26411.mRNA.1	178 –209	HHHHHHHHH+++HHHHHHHHHHHHH++++++	Invalid
DIPPA_21161.mRNA.1	543 –577	HHHHHHHHHHHHH+++HHHH+HH+HHHH+++++++	Valid
DIPPA_11950.mRNA.1	76 –106	HHHHHHHHHHHH++++HHHHHHHHHHHH+++++++	Valid

[a] Structures are considered valid if the following criteria apply: both the N-terminal and C-terminal helices must contain ≥7 helix-forming residues; non-helical amino acids are allowed inside helices; and three consecutive 'turn' residues must be present at positions 11 to 13 (see Methods).

References

1. Valach M, Moreira S, Petitjean C, Benz C, Butenko A, Flegontova O, Nenarokova A, Prokopchuk G, Batstone T, Lapébie P *et al*: **Recent expansion of metabolic versatility in *Diplonema papillatu*m, the model species of a highly speciose group of marine eukaryotes**. *BMC Biol* 2023, **21**(1):99.
2. Kaur B, Záhonová K, Valach M, Faktorová D, Prokopchuk G, Burger G, Lukeš J: **Gene fragmentation and RNA editing without borders: eccentric mitochondrial genomes of diplonemids**. *Nucleic Acids Res* 2020, **48**(5):2694-2708.
3. Lurin C, Andrés C, Aubourg S, Bellaoui M, Bitton F, Bruyère C, Caboche M, Debast C, Gualberto J, Hoffmann B *et al*: **Genome-wide analysis of Arabidopsis pentatricopeptide repeat proteins reveals their essential role in organelle biogenesis**. *Plant Cell* 2004, **16**(8):2089-2103.
4. McCall KA, Huang C, Fierke CA: **Function and mechanism of zinc metalloenzymes**. *J Nutr* 2000, **130**(5S Suppl).
5. Zastrow ML, Pecoraro VL: **Designing hydrolytic zinc metalloenzymes**. *Biochemistry* 2014, **53**(6):957-978.
